# mtDNA depletion confers specific gene expression profiles in human cells grown in culture and in xenograft

**DOI:** 10.1186/1471-2164-9-521

**Published:** 2008-11-03

**Authors:** Darren Magda, Philip Lecane, Julia Prescott, Patricia Thiemann, Xuan Ma, Patricia K Dranchak, Donna M Toleno, Krishna Ramaswamy, Kimberly D Siegmund, Joseph G Hacia

**Affiliations:** 1 Pharmacyclics Inc., 995 East Arques Avenue, Sunnyvale, CA, 94085, USA; 2 Department of Biochemistry and Molecular Biology, University of Southern California, 2250 Alcazar Street, IGM 240, Los Angeles, CA, 90089, USA; 3 Department of Preventive Medicine, University of Southern California, Los Angeles, CA, 90089, USA

## Abstract

**Background:**

Interactions between the gene products encoded by the mitochondrial and nuclear genomes play critical roles in eukaryotic cellular function. However, the effects mitochondrial DNA (mtDNA) levels have on the nuclear transcriptome have not been defined under physiological conditions. In order to address this issue, we characterized the gene expression profiles of A549 lung cancer cells and their mtDNA-depleted ρ^0 ^counterparts grown in culture and as tumor xenografts in immune-deficient mice.

**Results:**

Cultured A549 ρ^0 ^cells were respiration-deficient and showed enhanced levels of transcripts relevant to metal homeostasis, initiation of the epithelial-mesenchymal transition, and glucuronidation pathways. Several well-established HIF-regulated transcripts showed increased or decreased abundance relative to the parental cell line. Furthermore, growth in culture versus xenograft has a significantly greater influence on expression profiles, including transcripts involved in mitochondrial structure and both aerobic and anaerobic energy metabolism. However, both *in vitro *and *in vivo*, mtDNA levels explained the majority of the variance observed in the expression of transcripts in glucuronidation, tRNA synthetase, and immune surveillance related pathways. mtDNA levels in A549 xenografts also affected the expression of genes, such as *AMACR *and *PHYH*, involved in peroxisomal lipid metabolic pathways.

**Conclusion:**

We have identified mtDNA-dependent gene expression profiles that are shared in cultured cells and in xenografts. These profiles indicate that mtDNA-depleted cells could provide informative model systems for the testing the efficacy of select classes of therapeutics, such as anti-angiogenesis agents. Furthermore, mtDNA-depleted cells grown culture and in xenografts provide a powerful means to investigate possible relationships between mitochondrial activity and gene expression profiles in normal and pathological cells.

## Background

The discovery that mammalian cells grown in the presence of inhibitors of mitochondrial DNA (mtDNA) replication and transcription can lose all or almost all their mtDNA has provided a powerful means to study interactions between the mitochondrial and nuclear genomes, reviewed in [1-4]. The human mitochondrial genome is 16,569-bp in length and encodes 12S and 16S rRNAs, 22 tRNAs, and 13 polypeptides involved in the respiratory electron transport chain and ATP synthesis. The comprehensive MitoP2 database of human mitochondrial proteins    lists 870 human mitochondrial proteins encoded by the nuclear genome [5]. Proteomic and systems biology approaches will likely unearth additional proteins directly involved in human mitochondrial function [6]. However, fundamental questions will remain about the influences mtDNA has on the regulation of nuclear genes *in vivo*.

mtDNA-depleted ρ^0 ^cell lines are auxotrophic for pyruvate and uridine, and are incapable of aerobic respiration due to the lack of key respiratory chain components [7,8]. Nonetheless, mtDNA-depleted cell lines possess distorted mitochondria-like structures comprised of nuclear-encoded mitochondrial proteins [9-11] and demonstrate increased invasive behavior in culture [12-17] Furthermore, mtDNA-depleted cells can grow as tumor xenografts in immune-deficient mice in some instances [18,19], but not others [20].

Here, we generated mtDNA-depleted A549 ρ^0 ^lung cancer cells and validated their genetic and biochemical properties in culture and tumor xenografts. Our gene expression profiling analyses of parental A549 and A549 ρ^0 ^cells uncovered differential expression of transcripts relevant to immune recognition, autophagy of defective mitochondria, HIF-mediated gene regulation, the epithelial-mesenchymal transition (EMT), and elimination of lipophilic molecules. Furthermore, growth in culture versus in xenograft had a greater effect on the A549 transcriptome, especially nuclear-encoded genes important for mitochondrial function and energy metabolism, than the presence of mtDNA. Overall, mtDNA-depleted cells grown in culture and in xenograft provide complementary means to identify relationships between mitochondrial activity and cellular gene expression profiles that could serve as potential biomarkers of disease.

## Methods

### Cells and cell culture reagents

A549 lung cancer cells were obtained from the American Type Culture Collection. Unless otherwise indicated, all cell culture reagents were purchased from Invitrogen. mtDNA-deficient A549 ρ^0 ^cell cultures were prepared by serial passage of parental A549 cells for 28 population doublings in standard growth media (RPMI 1640 medium supplemented with 20 mM HEPES, 2 mM L-glutamine, 10% heat inactivated fetal bovine serum (Hyclone), 200 U/mL penicillin, and 200 μg/mL streptomycin) in the presence of ethidium bromide (100 ng/mL), sodium pyruvate (1 mM), and uridine (50 μg/mL). The glucose concentration in the standard growth media is approximately 11 mM. All subsequent experiments employing A549 and A549 ρ^0 ^cell cultures were conducted in the same standard growth media supplemented only with sodium pyruvate (1 mM) and uridine (50 μg/mL). Growth curves were obtained by Coulter counting. All experiments were conducted using cell culture populations and not purified clones.

### Estimation of mtDNA levels

mtDNA levels in A549 or A549 ρ^0 ^cell cultures were estimated using the pseudogene ratioing method [21]. Briefly, total DNA (both mitochondrial and genomic) was extracted from A549 or A549 ρ^0 ^cell cultures using the QIAamp DNA Mini Kit (Qiagen). These served as templates for PCR amplification using primers designed to anneal either to mtDNA or a previously described DNA-embedded mtDNA pseudogene (mtDNAψ) [22]. Amplicons derived from either nuclear DNA-embedded mtDNA pseudogene or mtDNA templates are predicted to be approximately the same length, but differ in sequence. These amplicons served as templates for a nested PCR using primers designed to amplify both the mtDNA-encoded *MT-CO2 *and the nuclear DNA-embedded mtDNAψ sequences. The resulting amplicons were subcloned using the StrataClone™ PCR cloning system (Stratagene). Plasmid DNA from individual colonies was prepared for sequencing using the illustra TempliPhi 100/500 DNA Amplification Kit (GE Healthcare). The resulting products were sequenced using BigDye Terminator v3.0 from Applied Biosystems diluted with halfBD (Genetix Ltd.), according to manufacturer's recommendations. We obtained sequence information from 70 colonies derived from A549 DNA templates (all showed mtDNA sequences) and 92 colonies derived from A549 ρ^0 ^DNA templates (all showed mtDNAψ sequences). mtDNA abundance in the original A549 or A549 ρ^0 ^DNA preparation was estimated based on the number of colonies showing mtDNA or mtDNAψ sequences, as previously described [21].

### Oxygen consumption

Oxygen consumption rates were measured using a Clark-type electrode [23]. In brief, plateau phase cultures of A549 or A549 ρ^0 ^cells were treated with antimycin A (1 μg/mL) or control vehicle for 3 hours. After medium was removed, cells were washed with Hanks balanced salt solution (HBSS), and harvested using trypsin/EDTA. Cells were resuspended in complete medium, stored on ice, and aliquots were removed for Coulter counting and viability (defined as ability to exclude propidium iodide). Prior to measurement, an aliquot of cell suspension (ca. 1 × 10^7 ^cells/mL) was incubated at 37°C for 2 minutes, and then transferred to the sealed electrode chamber of an Oxytherm apparatus (Hansatech, Norfolk, UK) to measure dissolved oxygen concentration. Background oxygen consumption by the electrode in the absence of cells was subtracted from all measurements.

### Quantitative RT-PCR analysis

RNA was obtained from cultured A549 cells or harvested from tumor specimens as described [24]. Total RNA was quantified using the Ribogreen RNA quantitation kit (Molecular Probes). One-step RT-PCR assays were set up in triplicate according to manufacturer's instructions using the Taqman master mix (Applied Biosystems, Inc.) and 50 ng of total RNA as template. The amount of RNA in each well was re-quantified and used for normalizations. Gene expression assay probe sets for *β-actin*, *MT-ATP6*, and *MT-CYB *were purchased from Applied Biosystems and assays were performed on an ABI PRISM 7300 instrument according to standard protocols.

### ELISA and Western blotting

Total HIF-1a protein was detected by sandwich ELISA using the DuoSet IC™ HIF-1a ELISA kit obtained from R&D Systems. Briefly, 96-well plates were coated with HIF-1a capture antibody overnight prior to blocking with 5% BSA in wash buffer. Protein lysates (50 μg protein per well prepared according to the manufacturer's instructions) were added for 2 hours, whereupon plates were washed and a biotinylated detection antibody specific for HIF-1a was added. A streptavidin-horseradish peroxidase format was used for detection. The optical density at 450 minus 570 nm was measured using a microplate reader (SpectraMax Plus). HIF-1a concentrations were calculated by linear regression using a standard curve prepared from HIF-1a standard supplied with the ELISA kit.

Western blotting was performed using antibodies against GLUT1 (aka SLC2A1, rabbit polyclonal), PGK1 (goat polyclonal), MTCO2 (mouse monoclonal), PDK1 (rabbit polyclonal), and DDIT4 (rabbit polyclonal) obtained from Santa Cruz Biotechnology, Molecular Probes, Stressgen, and Proteintech, respectively. All membranes were blotted with an anti-Hsc70 (mouse monoclonal, Santa Cruz Biotechnology) antibody to control for loading and transfer. HRP-conjugated goat anti-rabbit and goat anti-mouse secondary antibodies were from Pierce. HRP-conjugated donkey anti-goat secondary antibody was from Santa Cruz Biotechnology. HRP substrate was from Pierce. Bands were imaged and quantified in the linear range and normalized to Hsc70, using the ChemiDoc XRS Imaging System (BioRad Laboratories, Inc.).

### Gene expression profiling in cell culture

A549 or A549 ρ^0 ^human lung cancer cells (1 × 10^5 ^cells per T-25 flask in 7 mL complete RPMI 1640 medium) were seeded 8 days prior to RNA isolation. Each experiment was performed in triplicate. After incubation, all cultures were washed twice with HBSS supplemented with 0.5% BSA and total RNA was isolated and subjected to analysis on Human Genome U133A Arrays (Affymetrix), as described [24,25]. ArrayAssist software (Stratagene) was used to generate scaled log2 transformed gene expression scores based on the RMA algorithm and to conduct hierarchical clustering analyses, as previously described [24]. Probe sets with a > 1.5-fold differential expression (two-tailed Student's t-test *P *< 0.01 subjected to Benjamini and Hochberg correction for multiple comparisons, and > 20 unit difference in the geometric mean expression scores) in a comparison of interest are reported.

We used analysis of variance to estimate the proportion of variance explained by mtDNA status and explained by growth conditions, in the gene expression profiles from A549 ρ^0 ^and parental A549 cells grown in culture and in xenografts. Statistical significance is determined using the Bayes Moderated F test [26] implemented using the limma package in Bioconductor [27]. This uses an empirical Bayes approach, providing more stable inference when the number of arrays is small. *P*-values are adjusted for multiple comparisons using the Benjamini and Hochberg approach to control the false-discovery rate [28].

GeneOntology and KEGG analyses were conducted using WebGestalt software [29-31]. All scaled fluorescent intensity values and .cel files are available at the National Center for Biotechnology Information (NCBI) Gene Expression Omnibus (GEO) repository  under Series Accession Number GSE10957. In addition, all scaled fluorescent intensity values are available in Supplemental Table 1.

**Table 1:** Transcripts where 90% of the variance in gene expression is explained by mtDNA status

	Affymetrix Probe ID	Gene Symbol	Entrez GeneID	Gene Description	ρ^0 ^Proportion of Variance^a^	F test^b^	ρ^0 ^Vitro/A549 Vitro		ρ^0 ^Vivo/A549 Vivo	
							FC^c^	*P*^d^	FC^c^	*P*^d^
**Up-regulated in ρ^0 ^cells**	214247_s_at	*DKK3*	27122	dickkopf homolog 3	0.98	8.62 × 10^-11^	4.4	0.0009	3.7	0.0005
	209822_s_at	*VLDLR*	7436	very low density lipoprotein receptor	0.98	7.05 × 10^-10^	3.3	0.0057	2.7	0.0002
	210105_s_at	*FYN*	2534	FYN oncogene	0.97	9.85 × 10^-9^	3.4	0.0082	2.7	0.0009
	221305_s_at	*UGT1A8*	54576	UDP glucuronosyltransferase 1 family	0.97	1.21 × 10^-8^	8.3	0.0088	13.1	0.0007
	202242_at	*TSPAN7*	7102	tetraspanin 7	0.96	3.45 × 10^-11^	9.3	0.0046	9.3	0.0005
	205119_s_at	*FPR1*	2357	formyl peptide receptor 1	0.96	3.82 × 10^-9^	3.8	0.0045	4.8	0.0010
	208596_s_at	*UGT1A10*	54575	UDP glucuronosyltransferase 1 family	0.96	1.20 × 10^-10^	6.2	0.0051	10.2	0.0001
	219093_at	*PID1*	55022	hypothetical protein FLJ20701	0.95	1.41 × 10^-8^	5.4	0.0090	4.2	0.0020
	209040_s_at	*PSMB8*	5696	proteasome subunit	0.95	2.73 × 10^-10^	3.2	0.0048	2.9	0.0003
	206094_x_at	*UGT1A6*	54578	UDP glucuronosyltransferase 1 family	0.95	6.79 × 10^-11^	5.9	0.0041	9.7	0.0001
	219959_at	*PTHLH*	55034	parathyroid hormone-like hormone	0.94	2.77 × 10^-9^	2.8	0.0079	3.1	0.0005
	201042_at	*TGM2*	7052	transglutaminase 2	0.94	1.64 × 10^-6^	1.6	0.0245	1.6	0.0014
	207126_x_at	*UGT1A4*	54657	UDP glucuronosyltransferase 1 family	0.93	3.45 × 10^-11^	6.7	0.0048	11.5	0.0000
	203113_s_at	*EEF1D*	1936	eukaryotic translation elongation factor	0.92	1.10 × 10^-5^	1.5	0.0291	1.6	0.0042
	204802_at	*RRAD*	6236	Ras-related associated with diabetes	0.92	9.09 × 10^-7^	2.5	0.0289	2.7	0.0044
	215125_s_at	*UGT1A10*	54575	UDP glucuronosyltransferase 1 family	0.92	8.62 × 10^-11^	7.2	0.0045	12.7	0.0001
	213587_s_at	*ATP6V0E2*	155066	ATPase, H+ transporting V0 subunit e2	0.92	3.60 × 10^-6^	1.7	0.0447	1.9	0.0021
	217739_s_at	*PBEF1*	10135	pre-B-cell colony enhancing factor 1	0.91	4.54 × 10^-7^	3.8	0.0141	11.0	0.0004
	204532_x_at	*UGT1A4*	54657	UDP glucuronosyltransferase 1 family	0.91	3.96 × 10^-10^	5.8	0.0055	10.9	0.0000
	212160_at	*XPOT*	11260	exportin, tRNA	0.91	1.52 × 10^-5^	1.6	0.0524	1.9	0.0015
	212307_s_at	*OGT*	8473	GlcNAc transferase	0.91	6.52 × 10^-7^	1.7	0.0273	2.4	0.0007
	203262_s_at	*FAM50A*	9130	family with sequence similarity 50	0.91	3.01 × 10^-5^	1.5	0.0435	1.6	0.0030
	217738_at	*PBEF1*	10135	pre-B-cell colony enhancing factor 1	0.91	1.76 × 10^-7^	4.5	0.0054	11.2	0.0007
	201462_at	*SCRN1*	9805	secernin 1	0.90	2.79 × 10^-5^	1.4	0.0186	1.5	0.0032
	219762_s_at	*RPL36*	25873	ribosomal protein L36	0.90	8.04 × 10^-6^	1.4	0.0073	1.7	0.0014
	212909_at	*LYPD1*	116372	LY6/PLAUR domain containing 1	0.90	1.04 × 10^-5^	2.5	0.0069	1.7	0.0050
	215025_at	*NTRK3*	4916	neurotrophic tyrosine kinase, receptor	0.90	6.53 × 10^-7^	2.6	0.0179	2.2	0.0048
	201474_s_at	*ITGA3*	3675	integrin, alpha 3	0.90	1.57 × 10^-6^	1.8	0.0491	1.8	0.0018
**Down-regulated in ρ^0 ^cells**	211600_at	*MT-ND5*	4540	mito. encoded NADH dehydrogenase 5	0.99	4.48 × 10^-14^	-73.7	0.0007	-46.9	0.0001
	205674_x_at	*FXYD2*	486	FXYD domain ion transport regulator 2	0.98	3.45 × 10^-11^	-11.0	0.0029	-14.8	0.0004
	211203_s_at	*CNTN1*	1272	contactin 1	0.97	4.65 × 10^-9^	-4.7	0.0009	-3.6	0.0015
	204698_at	*ISG20*	3669	interferon stimulated exonuclease gene	0.96	3.67 × 10^-8^	-3.5	0.0056	-2.9	0.0014
	210065_s_at	*UPK1B*	7348	uroplakin 1B	0.95	4.17 × 10^-8^	-4.3	0.0071	-6.2	0.0013
	203108_at	*GPRC5A*	9052	G protein-coupled receptor	0.95	4.96 × 10^-9^	-3.4	0.0048	-5.9	0.0001
	207434_s_at	*FXYD2*	4151	FXYD domain ion transport regulator 2	0.95	2.29 × 10^-11^	-9.8	0.0063	-12.9	0.0003
	201065_s_at	*GTF2I*	2969	general transcription factor II, i	0.95	6.30 × 10^-7^	-2.0	0.0109	-1.7	0.0010
	210064_s_at	*UPK1B*	7348	uroplakin 1B	0.95	1.51 × 10^-8^	-5.8	0.0081	-5.9	0.0020
	222088_s_at	*SLC2A3*	6515	solute carrier family 2, member 3	0.95	1.46 × 10^-8^	-2.1	0.0080	-2.4	0.0010
	33304_at	*ISG20*	3669	interferon stimulated exonuclease gene	0.95	3.70 × 10^-7^	-2.9	0.0077	-2.4	0.0036
	218995_s_at	*EDN1*	1906	endothelin 1	0.94	3.16 × 10^-7^	-2.2	0.0333	-2.7	0.0014
	219045_at	*RHOF*	54509	ras homolog gene family, member F	0.93	4.81 × 10^-7^	-2.9	0.0062	-2.0	0.0018
	214774_x_at	*TNRC9*	27324	trinucleotide repeat containing 9	0.93	4.39 × 10^-7^	-2.9	0.0109	-3.9	0.0027
	206291_at	*NTS*	4922	neurotensin	0.93	6.81 × 10^-8^	-102	0.0009	-17.9	0.0011
	201909_at	*RPS4Y1*	6192	ribosomal protein S4, Y-linked 1	0.92	3.55 × 10^-8^	-5.8	0.0071	-18.8	0.0000
	206700_s_at	*JARID1D*	8284	jumonji, AT rich interactive domain 1D	0.90	2.41 × 10^-7^	-2.3	0.0366	-4.0	0.0000
	203043_at	*ZBED1*	9189	zinc finger, BED-type containing 1	0.90	6.01 × 10^-6^	-1.7	0.0427	-1.9	0.0026
	202499_s_at	*SLC2A3*	6515	solute carrier family 2, member 3	0.90	2.86 × 10^-6^	-1.9	0.0128	-2.3	0.0073
	216623_x_at	*TNRC9*	27324	trinucleotide repeat containing 9	0.90	5.79 × 10^-7^	-2.6	0.0128	-3.5	0.0038

^a^Proportion of variance in gene expression due to mtDNA status in cultured cells and xenografts

^b^Bayes modified F test (multiple hypothesis corrected)

^c^Fold-change

^d^Benjamini and Hochberg corrected two-tailed Student's t-test

### Gene expression profiling in mouse xenograft models

Animal care was in accordance with NIH and institutional guidelines. For the A549 xenograft model, 1.7 million A549 cells were injected subcutaneously/intramuscularly into the right hind flank of 6 week old CD-1 nude mice that had been irradiated with 4 Gy of total body irradiation from a ^137^Cs radiation source one day prior to tumor implantation [32]. For the A549 ρ^0 ^xenograft model, 1.5 million cells were used. Tumor and body weight measurements (6 or 7 mice per group) were performed three times per week once tumors became palpable. This occurred 41 versus 9 days post-implantation in ρ^0 ^and parental A549 xenograft models, respectively. Tumor volume was calculated using the equation V (mm^3^) = a × b^2^/2, where a is the largest diameter and b is the smallest diameter. No significant body weight loss was observed. To perform gene expression profiling, tumors (4 tumors per group) were harvested and snap frozen immediately on dry ice when the tumor size reached 500–800 mm^3^. Tumor tissue was dissected, homogenized in Trizol, and total RNA was isolated and subjected to gene expression analysis as described above.

### Additional Files

Additional information about the data reported in this manuscript, including formatted gene expression data in these studies are available at *BMC Genomics*.

## Results and discussion

### Establishment of A549 ρ^0 ^cell cultures

**Figure 1:**
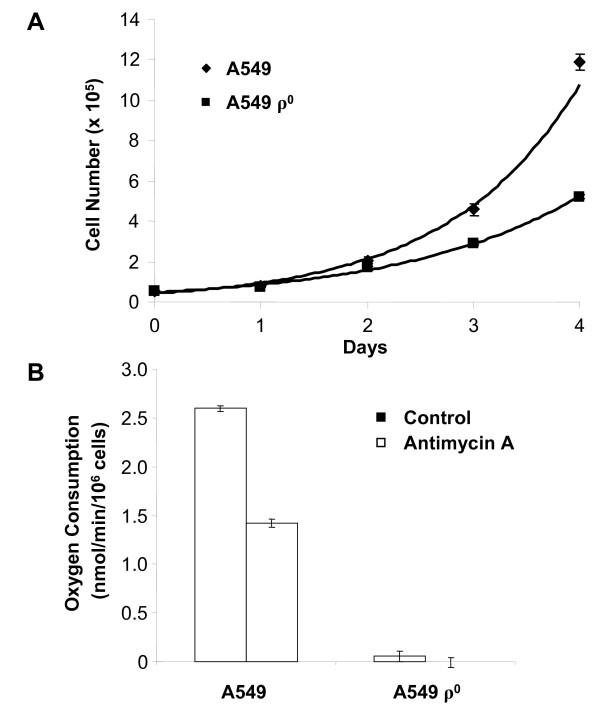
**Characterization of cultured A549 and A549 ρ^0 ^cells**. (*A*) Growth rates of A549 and A549 ρ^0 ^cells in culture are provided. Experiments were run in triplicate with error bars representing ± 1 standard deviation. Doubling times obtained from the fitted curves (N = N_0_e^rt^) were 20.4 and 27.3 hours for parental A549 and A549 ρ^0 ^cultures, respectively. (*B*) The oxygen consumption of A549 and A549 ρ^0 ^cultured cells is provided. Where indicated, cells were treated with 1 μg/mL antimycin A, an inhibitor of the mitochondrial electron transport chain (ETC). Experiments were run in triplicate with error bars representing ± 1 standard deviation.

Candidate A549 ρ^0 ^lung cancer cell cultures were prepared using previously described ethidium bromide-based protocols [12]. Consistent with prior reports of ρ^0 ^cell lines (e.g. [7,9,33]), the resulting cells displayed auxotrophic growth dependence on pyruvate and uridine and elongated shape (data not shown). The growth rate of the candidate ρ^0 ^cells (27 hour generation time) was slower than the parental line (20 hour generation time) in pyruvate and uridine supplemented medium (Fig. 1A).

We used the recently described pseudo-gene ratioing technique to estimate the amount of mtDNA remaining in the candidate A549 ρ^0 ^cell cultures (Methods) [21]. This technique is based on quantifying the relative co-amplification of a mtDNA sequence and a nuclear-embedded mitochondrial pseudogene (mtDNAψ) sequence [22] using total DNA extracted from candidate A549 ρ^0 ^cells and parental A549 cells. Assuming the mtDNAψ sequence is present in two copies per candidate A549 ρ^0 ^cell, we could estimate with 95% confidence that the candidate A549 ρ^0 ^cell cultures contained less than one copy of mtDNA per 14 cells. As a positive control, we used the same technique to estimate that a minimum of 45 mtDNA copies were extracted from each parental A549 cell (exact binomial upper 95% confidence limit). As a consequence of these observations, as well other biochemical characterizations described below, we will hereafter refer to the candidate A549 ρ^0 ^cell cultures as A549 ρ^0^cells.

### Cultured A549 ρ^0 ^cells have impaired rates of oxygen consumption

To characterize mitochondrial function in the parental A549 and A549 ρ^0 ^cells, we compared their rates of oxygen consumption. As expected, parental A549 cells showed robust oxygen consumption while the oxygen consumption of the A549 ρ^0 ^cells was indistinguishable (P = 0.41) from background (Fig. 1B). To demonstrate the involvement of respiration, we also treated parental A549 and A549 ρ^0 ^cells with antimycin A, an inhibitor of mitochondrial complex III of the electron transport chain (ETC) [34], prior to measuring oxygen consumption. As expected, antimycin A-treated A549 cells showed reduced oxygen consumption, while A549 ρ^0 ^cells were not significantly affected by drug-treatment (Fig. 1B). These results strongly suggest that residual mitochondria-dependent aerobic respiration in A549 ρ^0 ^cells, if present, is below the threshold that can be measured in this assay.

### A549 ρ^0 ^cells grow in tumor xenografts at a slower rate than their parental cells

**Figure 2:**
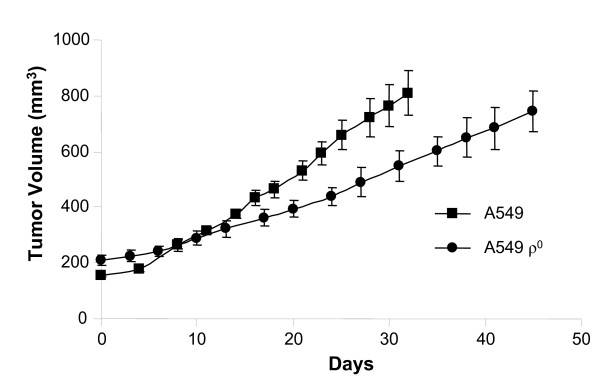
**Growth rates of A549 and A549 ρ^0 ^xenografts**. Median tumor volume over time in nude mice bearing tumors derived from A549 or A549 ρ^0 ^cells. Tumor volume measurements commenced at an initial average tumor volume of 155 mm^3 ^and 208 mm^3^, respectively. Experiments were conducted using six or seven mice per cohort with error bars representing ± 1 standard error of the mean (SEM).

Parental A549 cells and their ρ^0 ^derivatives both grew as xenografts when implanted shallowly (subcutaneously/intramuscularly) in the flank muscle of nude mice. mtDNA-depleted cell lines have been reported to form xenograft tumors [13,18,19]. Here, we found that A549 ρ^0 ^cells grow readily in xenograft, although at a slower rate and after a longer induction period (approximately 30 days) relative to the parental line (Fig. 2). This is consistent with the growth characteristics of tumor xenografts derived from N2B ρ^0 ^osteosarcoma [18], E3 ρ^0 ^serous ovarian carcinoma [18], HSA ρ^0 ^cervical cancer [18], and T47D ρ^0 ^breast cancer [19] cell lines relative to their parental counterparts.

### A549 ρ^0 ^cells do not express mtDNA-derived transcripts in culture or in xenografts

We performed quantitative PCR analysis of mtDNA-encoded *MT-ATP6 *and *MT-CYB *transcripts in A549 and A549 ρ^0 ^cells grown in culture and in xenografts, in the absence of ethidium bromide. In contrast to the parental A549 cells, the expression of these two transcripts could not be detected in ρ^0 ^cells (Additional File 1). This strongly suggests that, in bulk, the A549 ρ^0 ^cells retained their mtDNA-depleted genotype over the course of their growth in culture and in xenograft. This was important to ascertain since it was possible that during their growth in xenograft, rare mtDNA-positive cells present in the injected A549 ρ^0 ^cell population could have substantially increased in abundance and affected our downstream analyses. Similarly, it is possible that A549 or A549 ρ^0 ^cells with certain genetic or epigenetic changes (e.g. those that confer a growth advantage) were selected for in culture or in xenografts and affected our results. This should be considered when interpreting the molecular analyses discussed below.

### Gene expression profiling of cultured cells

Gene expression profiles were generated in plateau phase A549 and A549 ρ^0 ^cells cultured in fresh medium supplemented with pyruvate and uridine prior to RNA isolation (see Additional File 2 for all data and Additional File 3 for volcano plot). All 276 transcripts that were differentially expressed (defined for every pair-wise comparison discussed hereafter as being > 1.5-fold change, two-tailed Student's t-test *P *< 0.01 (subjected to Benjamini and Hochberg correction for multiple comparisons), and > 20 unit difference in geometric mean expression scores) are listed in Additional File 4. These include 131 transcripts that were more highly expressed in A549 ρ^0 ^cells and 145 that were more highly expressed in A549 cells.

### Increased levels of EMT-related transcripts in cultured A549 ρ^0 ^cells

To analyze differentially expressed genes in A549 cells and their mtDNA-depleted ρ^0 ^counterparts, we conducted Gene Ontology (GO) analyses on transcripts that were more highly expressed in the A549 ρ^0 ^cells than the parental cell line (see Additional File 5A for transcript names and Additional File 2 for data analysis of specific transcript). We used stringent criteria (Fisher's exact test *P *< 0.001 and at least 4 transcripts) for selection of enriched categories. The enriched categories included those related to cell adhesion (15 transcripts) and cell motility (4 transcripts). Upon further inspection of the transcripts in these categories, it became apparent that they reflect transcriptional signatures of the epithelial-mesenchymal transition (EMT), a developmental process by which stationary epithelial cells transform into mobile mesenchymal cells [35]. EMT can be driven by the activity of the transcription factor *SNAI2*, positively regulated by *LOXL2 *[36], which leads to the over-expression of *CDH2*, *ITGB3*, and *VIM*. All five genes are over-expressed in the ρ^0 ^cells. The fact that *CDH1 *and *FGA *are also less abundant in the ρ^0 ^cells further supports the hypothesis that the ρ^0 ^cells show an increasingly mesenchymal phenotype relative to their parental counterparts [35]. The above observations are consistent with prior reports of mtDNA-depleted A549 cells showing increased invasive behavior relative to parental A549 cells in a Matrigel basement membrane matrix invasion assay system [12].

### Increased metal ion binding and glucuronidation gene expression in A549 ρ^0 ^cell cultures

Two enriched GO categories were related to metal ion binding (*LOXL2 *and metallothionein family members *MT1H*, *MT1G*, *MT1X*, and *MT2A*) and glucuronidation (including UDP glucuronosyltransferase 1 family members). The increased expression of MTF-1 regulated genes (i.e. metallothioneins) could indicate higher baseline levels of oxidative stress and/or free metals in the ρ^0 ^cells relative to their parental counterparts [25]. However, it is noteworthy that several oxidative stress response genes (e.g. *GPX2*, *GPX3*, and *GLRX*) are under-expressed in these ρ^0 ^cells. Glucuronidation is a cellular metabolic pathway in which UDP-glucuronosyltransferases (UGTs) convert lipophilic compounds, both endogenous and xenobiotic, into more readily excreted polar products. UGTs catalyze the transfer of the glucuronate moiety of uridine diphosphoglucuronate (UDPGA) to specific substrates, in order to attain this increased polarity. The observed differentially expressed UGT1A family members have different substrate specificities and collectively can react with a variety of endogenous lipophilic molecules, such as fatty acids and steroids [37-41]. Given that ρ^0 ^skin fibroblasts have impaired mitochondrial fatty acid β-oxidation [42], we propose that the increased abundance of glucuronidation-related transcripts in A549 ρ^0 ^cells is a survival mechanism to remove excess fatty acids that are normally metabolized to carbon dioxide and water in functional mitochondria.

### Expression profiles of HIF-1 responsive transcripts in A549 ρ^0 ^cells

Next, we focused on identifying key transcription factors that could account for a significant number of over-expressed transcripts in A549 ρ^0^cells. mtDNA-deficient cells have proven useful for dissecting the role that mitochondria play in HIF-mediated responses to oxygen levels (reviewed by [43]). In fact, increased baseline levels of HIF-1 activity in cultured ρ^0 ^cell lines have been noted by others [44,45]. In our A549 ρ^0 ^cells, HIF-1 appeared to be an excellent candidate given the over-expression of two well-established downstream genes (*VEGFA *and *BNIP3*). We began our analyses by focusing on a group of 95 probe sets representing 63 unique HIF-1 responsive genes highlighted in a recent comprehensive review [46] (Additional File 6). While no other HIF-1 responsive genes reached our statistical criteria for over-expression, four other well-established HIF-1 regulated transcripts (*IGFBP1*, *IGFBP3*, *TF*, and *PTGS2*) were less abundant in the ρ^0 ^cells relative to their parental cells. This could reflect the influence of other transcription factors or accessory proteins that regulate HIF-1 activity.

The mechanism underlying the differential expression of HIF-regulated transcripts in mtDNA-deficient cells is unknown. For example, it has been proposed that a feedback loop involving mitochondrial enzymes and citric acid cycle intermediates leads to HIF-1 stabilization and tumorigenesis in several inherited forms of cancer caused by mtDNA mutations [47]. Another possible explanation might involve insulin-like growth factor signaling which has been shown to increase HIF-1 levels [48]. Importantly, the GO category insulin-like growth factor binding (*CYR61*, *HTRA1 *aka *PRSS11*, *IGFBP4*, and *IGFBP7*) was enriched for transcripts that were more abundant in the ρ^0 ^relative to parental cells (Additional File 5A).

### A549 ρ^0 ^cells over-express HIF-1 a protein relative to the parental cell line

**Figure 3:**
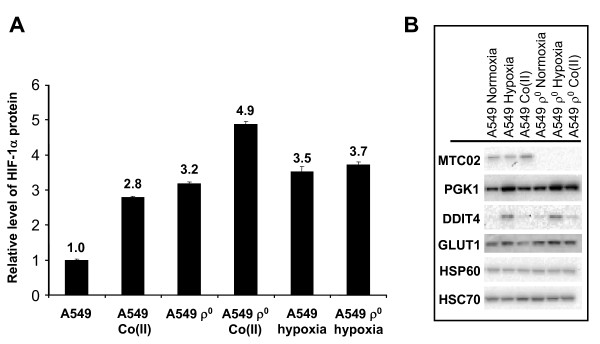
**Levels of HIF-1α and HIF-1 target proteins in A549 and A549 ρ^0 ^cells**. Plateau phase cultures were treated with control vehicle (mannitol), cobalt acetate (cobalt, 100 μM), or hypoxia (1.5% oxygen atmosphere). (*A*) Levels of HIF-1α protein relative to control vehicle after 4 hours treatment as measured by ELISA. Effect of incubation under hypoxic conditions or with cobalt acetate is shown for comparison. Error bars indicate one standard deviation. (*B*) Representative Western blot showing MT-CO2, PGK1, DDIT4, GLUT1, and HSP60 levels in A549 and A549 ρ^0 ^cells. PGK1 and DDIT4 levels were more abundant (1.4- and 4.0-fold, respectively) in A549 ρ^0 ^cells relative to A549 cells. In contrast, GLUT1 and HSP60 levels were only marginally changed (1.1- and 1.25-fold less abundant) in A549 ρ^0 ^cells relative to A549 cells. All estimates were made via phorphorimaging analysis with HSC70 levels serving as a loading control. Note that MT-CO2 levels could not be detected in any A549 ρ^0 ^experiment while DDIT4 was expressed at low levels, but could be quantified in normoxic cells.

To explore the mechanistic basis for the observed differential expression of HIF-responsive transcripts in culture, we measured the levels of HIF-1α protein in ρ^0 ^and parental cells by ELISA (Fig. 3A). The background levels of HIF-1α were found to be 3-fold higher in ρ^0 ^cells compared to the parental line. HIF-1α levels increased in the parental A549 line following treatment with cobalt acetate or incubation under hypoxic (1.5% O_2_) conditions. In A549 ρ^0 ^cells, HIF-1α levels were increased modestly following cobalt treatment but did not appear to be changed by hypoxic treatment. However, the level of HIF-1α protein does not solely determine its ability to induce gene expression, as post-transcriptional modifications are also known to modulate its activity [49].

To further explore the possible functional consequences of HIF-1 over-expression, we measured the levels of several HIF-regulated gene products by Western blot (Fig. 3B). The MT-CO2 product was included in this analysis to demonstrate the absence of this mtDNA-encoded protein in A549 ρ^0 ^cells. In accordance with the expression data, we found that PGK1 and DDIT4 protein levels were increased in A549 ρ^0 ^cells (Fig. 3B). However, GLUT1 (aka SLC2A1) protein levels were essentially unchanged in A549 and A549 ρ^0 ^cells despite the fact that its transcript was 2.0-fold more abundant (corrected *P *= 0.017) in A549 ρ^0 ^cells. As could be expected, the incubation of either parental or ρ^0 ^cells in the presence of cobalt or under hypoxic conditions led to increased levels of these HIF-regulated proteins. The effect of cobalt was not as significant as that of hypoxia under these conditions. These data demonstrate that although baseline HIF-1 activity is higher in ρ^0 ^than parental cells, HIF-regulated activity can be induced further in both cases.

### Decreased icosanoid metabolism and cytoskeleton gene expression in cultured A549 ρ^0 ^cells

In parallel, we conducted separate GO analyses on transcripts that were less abundant in A549 ρ^0 ^cells (*P *< 0.001 and at least four probes sets) (Additional File 5B). The blood pressure regulation (*FGB*, *PTGS2*, *FGG*, and *FGA*) and icosanoid metabolism (*AKR1C2*, *PLA2G4A*, *PTGS2*, and *MGST2*) GO categories were significantly enriched for such transcripts. The presence of multiple fibrinogen family members in the former category could reflect possible differences in cell adhesion in ρ^0 ^cells. The icosanoid metabolism category could relate to the role arachidonic acid plays in the regulation of steroidogenesis and mitochondrial membrane permeability. It has been proposed that ATP generated by oxidative phosphorylation plays a key role in the generation and export of arachidonic acid from the mitochondria [50]. Through its role in leukotriene metabolism, arachidonic acid mediates the activity of steroidogenic acute regulatory protein (StAR), a key regulator of steroidogenesis. In addition, arachidonic acid can uncouple oxidative phosphorylation and alter mitochondrial membrane permeability [51].

The intermediate filament GO category was enriched for transcripts that were less abundant in ρ^0 ^cells (i.e. *KRT8*, *KRT19*, *NEFL*, *KRT18*, and *DSP*). *KRT18 *plays a key role in maintaining the normal morphology of mitochondria [52]. Interestingly, *VIM*, another component of intermediate filaments shown to support mitochondrial morphology and organization [53], is over-expressed in ρ^0 ^cells. The dysregulation of intermediate filament genes in ρ^0 ^cells could contribute to their aberrant mitochondrial morphology and/or represent cellular responses to rescue mitochondrial organization.

### Increased tRNA synthetase, MHC Class I, and lysosome gene expression in A549 ρ^0 ^xenografts

Total RNA from A549 or A549 ρ^0 ^tumors (four per cohort) were also subject to gene expression profiling analysis (see Additional File 3 for volcano plot). All 948 transcripts that were differentially expressed are listed in Additional File 7. These include 485 transcripts that were more highly expressed in the A549 ρ^0 ^tumors and 463 that were more highly expressed in A549 tumors. Three major functional categories emerged from GO analyses of over-expressed genes in ρ^0 ^xenografts (Additional File 8A). These categories were related to tRNA aminoacylation (composed of 9 tRNA synthetases), MHC class I (*B2M*, *MR1*, and six HLA gene family members), and the lysosome (23 transcripts).

The transcriptional induction of tRNA synthetases was previously highlighted in the analysis of cultured ρ^0 ^and parental lymphoblasts [44]. Recently, there is increased evidence that tRNA synthetases have functions additional to joining specific amino acids to their cognate tRNAs. These include the regulation of angiogenesis and inflammation [54,55].

The over-expression of MHC Class I peptides is consistent with a report that MHC I is over-expressed in fibroblasts from patients with mtDNA defects as well as in cultured osteosarcoma ρ^0 ^cells relative to their parental counterparts [56]. In that study, IFN-γ treatment enhanced MHC1 over-expression in cultured osteosarcoma ρ^0 ^cells. Thus, the over-expression of interferon gamma receptor 1 (*INFGR1*) we observed in A549 ρ^0 ^xenografts (1.8-fold, corrected *P *= 0.004) could play a role in further stimulating MHC Class I peptide expression in these cells.

It has been proposed that MHC Class I over-expression in cells with mutated mitochondrial proteins provides a mechanism by which the immune system can recognize and eliminate defective cells [56]. Although ρ^0 ^cells do not have dysfunctional proteins encoded by mtDNA, the over-expression of MHC Class I genes may simply reflect a mechanism for marking cells with defective mitochondrial function.

The over-expression of lysosome genes could relate to their function in removing defective mitochondria within cells through autophagy [57]. This could complement the immune surveillance mediated removal of cells with defective mitochondria discussed above. We speculate that the over-expression of lysosome-related genes could reflect increased numbers of lysosomes and/or simply the increased functional activity of existing lysosomes.

### A549 ρ^0 ^xenograft transcriptomes indicate decreased cell proliferation and glycolysis

Mitosis (21 transcripts) and spindle organization and biogenesis (9 transcripts) were two major functional categories showing enrichment for down-regulated transcripts in A549 ρ^0 ^xenografts (Additional File 8B). This would be expected given the lower proliferation rate of the A549 ρ^0 ^cells relative to their parental counterparts (Fig. 1A). The enrichment in the actin binding (21 transcripts) and cytoskeleton protein binding (27 transcripts) categories could also reflect differences in growth rates. Alternatively, they could relate to altered mitochondrial morphology, as discussed in our analysis of cultured ρ^0 ^cells.

Surprisingly, A549 ρ^0 ^xenografts showed decreased expression in the glycolysis category (nine transcripts). This would appear to be counter-intuitive given the fact that these xenografts do not express mtDNA-derived transcripts (Additional File 1) or MT-COX2 protein (Fig. 3B) and thus should have severely impaired oxidative phosphorylation. Indeed, the 4.7-fold increased abundance of *PCK2 *in A549 ρ^0^cells could indicate higher levels of gluconeogenesis, consistent with prior reports in mtDNA-depleted A549 [12], 206B ρ^0^osteosarcoma [58], and ARPE19 ρ^0 ^retinal pigment epithelial [58] cultured cells. Apart from the aforementioned lower growth rate of ρ^0 ^xenografts, this probably reflects the general reduced abundance of HIF-regulated transcripts (*GAPDH*, *PKM2*, *ENO2*, *LDHA*, *EGLN1*, *SLC2A1*, *TF*, *P4HA1*, *IGF2*, *CA9*, *HIG2*, *ADM*, and *EGLN3*) as compared to the parental A549 xenografts (Additional File 6). This occurs despite the fact that HIF-1α is over-expressed (2.6-fold, *P *= 1.6 × 10^-4^) along with two other well-established HIF targets (*HK1 *and *BHLHB3*). As discussed earlier, this is consistent with the post-translational control of HIF-1 α activity (protein modification or subunit localization) and/or the activity of other transcription factors.

### Contribution of mtDNA to gene expression profiles of cultured cells and xenografts

**Figure 4:**
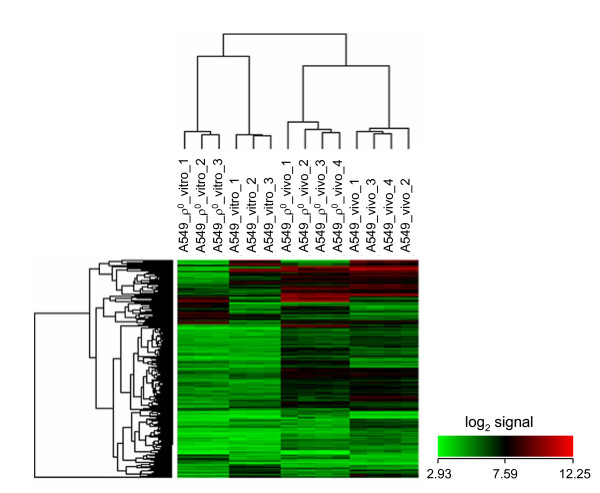
**Hierarchical clustering analysis of expression data from A549 and A549 ρ^0 ^cells in culture and in xenografts**. The dendrograms were generated based on average linkage hierarchical clustering of expression data from 538 probe sets whose coefficient of variation was greater than 0.10. Data from A549 cultures are provided in triplicate.

While gene expression differences between A549 cells grown in culture and in xenograft have been reported [59], comparison for both parental and mtDNA-deficient cells have not been previously performed. Interestingly, a greater number of transcripts were differentially expressed due to growth conditions (culture versus xenograft) than by mtDNA status. Importantly, infiltrating mouse cells have been demonstrated not to influence significantly gene expression profiles of tumor xenografts when using similar Affymetrix human microarrays [59]. All 2,512 transcripts that were differentially expressed in xenograft versus culture for the parental A549 line are listed in Additional File 9. The 2,108 differentially expressed transcripts for the A549 ρ^0 ^cells grown in xenograft versus culture are listed in Additional File 10 (see Additional File 3 for volcano plot). These results are borne out by hierarchical clustering analysis which first shows a clear separation of the gene expression profiles grown in culture versus xenograft and then separation according to mtDNA status, based on gene expression data obtained from 538 probe sets whose coefficient of variation was greater than 0.10 (Fig. 4).

We calculated the relative fraction of the variance associated with mtDNA status in the gene expression profiles from A549 ρ^0 ^and parental A549 cells grown in culture and in xenografts (Additional File 2). In particular, we focused on those transcripts for which over 50% of the variance (Benjamini and Hochberg corrected Bayes moderated F test *P *< 0.01) could be explained by mtDNA status and where there was significant differential expression in any two of the pair-wise comparisons of A549 ρ^0 ^and parental A549 cells grown in culture or in xenograft. Overall, we discovered 430 transcripts (229 up-regulated and 201 down-regulated in A549 ρ^0 ^relative to the parental A549 cells) that met these criteria (Additional File 11). Table 1 provides a listing of a subset of these transcripts where at least 90% of the variance in gene expression was dependent upon mtDNA status.

**Figure 5:**
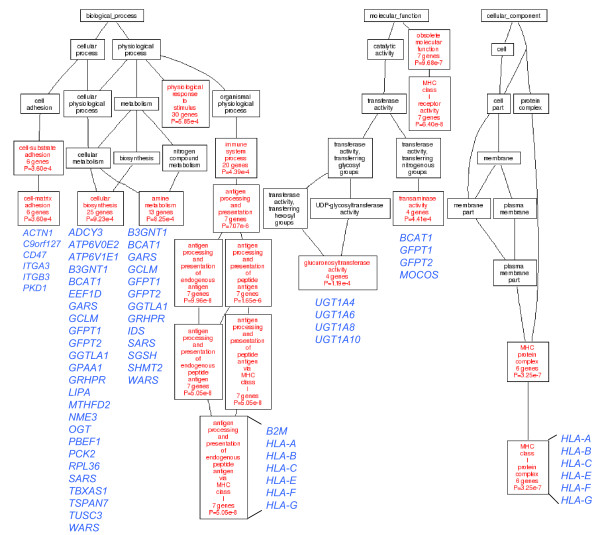
**Gene Ontology analysis of up-regulated transcripts in ρ^0 ^cells whose variance is strongly influenced by mtDNA status**. The directed acyclic graph (DAG) represents the relationships of categories (shown in red) that are enriched for transcripts where mtDNA status accounted for over 50% of the observed variance in gene expression and are more abundant *in vitro *(i.e. cell culture) and/or *in vivo *(i.e. xenograft) in A549 ρ^0 ^cells relative to parental A549 cells. All 229 probe sets used in this analysis are provided in Additional File 11. The names of genes in terminal categories are provided the blue.

Importantly, the probe set that was most strongly affected (in this case down-regulated) by mtDNA status measures the relative abundance of *MT-ND5*, the only mtDNA-encoded transcript represented in this microarray (Table 1). *MT-ND5 *is not expressed in the A549 ρ^0 ^cells in culture or in xenograft (i.e. ranking within the lowest 15^th ^percentile of all median gene expression scores in both cases). This provided us with empirical proof that our sorting strategy was sensitive to detecting transcripts dependent upon mtDNA status. Overall, we found that the variance in the expression of transcripts within MHC-1 (6 transcripts) and glucuronidation (4 transcripts) GO categories are all highly dependent upon mtDNA status (Fig. 5). These transcripts were more abundant in A549 ρ^0 ^relative to the parental A549 cells. The MHC-1 and glucuronosyltransferase categories were previously discussed as having significance for the immune surveillance of cells with effective mitochondria and the detoxification of lipids that can accumulate due to defects in fatty acid metabolism. On the other hand, transcripts related to the protein complex assembly (11 transcripts) and chromatin (7 transcripts) GO categories were highly dependent upon mtDNA status and less abundant in A549 ρ^0 ^cells relative to the parental A549 cells (Additional File 12). This could reflect their slower growth rates relative to the parental cell line.

In addition, mtDNA status affected multiple transcripts related to the above discussed epithelial-mesenchymal transition (EMT). Over 50% of the variance in the expression levels derived from probe sets for the previously described *SNAI2*, *ITGB3*, *CDH1*, and *FGA *genes could be explained by mtDNA status. The expression levels of all these genes indicated A549 ρ^0 ^cells have increased mesenchymal phenotypes relative to the parental A549 cells. However, the fold-changes in expression of these genes in ρ^0 ^versus parental cells were greater in culture than in the tumor xenografts (Additional File 2).

In contrast to genome-wide expression studies performed in yeast lacking mtDNA (ρ^0 ^petites), we observed no evidence for a strong dependence of the variance in peroxisomal gene expression (especially based on 26 probe sets for peroxin (*PEX*) genes responsible for peroxisome assembly) due to mtDNA status [60]. Nevertheless, this does not preclude mtDNA status affecting peroxisome function, through abundance and/or activity. For example, we observed that the mtDNA status had a significant effect on *PHYH *expression (explains 76% of the observed variance in expression, F-test *P *= 4.56 × 10^-4^). *PHYH *encodes a peroxisomal enzyme critical for the catabolism of phytanic acid, a branched chain fatty acid [61,62]. *AMACR*, which also encodes an enzyme critical for phytanic acid metabolism, is also expressed higher in A549 ρ^0 ^xenografts relative to A549 xenografts (2.0-fold, corrected *P *= 0.007). Interestingly, AMACR, which localizes to both peroxisomes and mitochondria, is an emerging biomarker for prostate cancer [63]. Given reports of frequent mitochondrial defects in prostate cancer [64], it is tempting to speculate that the over-expression of AMACR in prostate cancer could be related to defective mitochondrial function. However, it should be emphasized that the mitochondrial defects in our mtDNA-depleted A549 system will be more severe than those found in human tumors.

### mtDNA-dependent expression profiles show agreement with prior reports

It can be questioned whether the observed mtDNA-dependent gene expression profiles are (i) shared amongst all cell lines, (ii) unique to our system, or (iii) reflect possible mutations in acquired in the nuclear genome of the ethidium bromide-treated A549 ρ^0 ^cells. To begin to address these issues, we searched for previous reports of differential gene expression in cultured ρ^0 ^cells relative to their parental cell lines. It is important to note that there is a limited literature on gene expression profiling in mtDNA-depleted cell cultures [16,44,65,66] and that there are no previous reports of gene expression profiling in mtDNA-depleted xenografts. Nevertheless, we found confirmatory evidence for our observed major categories of mtDNA-dependent transcripts levels including: tRNA synthetases (over-expressed in osteosarcoma ρ^0 ^cells [66] and lymphoblastoid ρ^0 ^cells [44]), MHC-related transcript (over-expressed in osteosarcoma ρ^0 ^cells [56] and breast cancer ρ^0 ^cells [65]), UDP-glucuronosyltransferase (over-expressed in breast cancer ρ^0 ^cells [65]), and HIF1-α (over-expressed in lymphoblastoid ρ^0 ^cells [44]). Furthermore, the *FXY2D2 *(aka *ATP1G1*) and the *VLDR *transcripts, whose levels were strongly dependent upon mtDNA-status and over-abundant in A549 ρ^0 ^cells (Table 1), were found to be over-expressed in human fibroblasts with mutations in nuclear-encoded mitochondrial Complex I genes [67] and retinal pigment epithelial ρ^0 ^cells [14], respectively. Similarly, metallothionein transcripts were over-expressed in fibroblasts containing mutated nuclear-encoded mitochondrial genes, in agreement with our observations in A549 ρ^0 ^cell cultures [67]. Thus, results from multiple laboratories testing different ρ^0 ^cell cultures support the assertion that our current analyses at least partially reflect mtDNA-dependent gene expression relevant to multiple cell types. Future gene expression profiling experiments of mtDNA-depleted cells generated using non-chemical-based protocols, such as expressing siRNA against the *TFAM *gene [68] or a dominant negative form of the mitochondria-specific DNA polymerase-gamma [69], could provide valuable empirical data to assess our candidate mtDNA-dependent gene expression profiles. Ultimately, large-scale proteomic characterization of mtDNA-depleted and parental cells will be necessary to rigorously assess possible functional consequences of differential gene expression [15,70].

### Contribution of growth conditions to gene expression profiles

Next, we calculated the relative fraction of the variance associated with growth conditions in the gene expression profiles from ρ^0 ^and parental cells grown in culture and in xenografts. At first, we focused on those transcripts for which over 50% of the variance (corrected F test *P *< 0.01) could be explained by growth conditions and where there was differential expression in any of the pair-wise comparisons of ρ^0 ^and parental A549 cells grown in culture or in xenograft. Overall, we uncovered 2,633 transcripts that met these criteria. In order to reduce transcript complexity, we focused on the subset of 551 transcripts (316 up-regulated and 235 down-regulated in xenografts relative to cultured cells) where growth conditions contributed to a robust 90% of the variance observed in the data set (Additional File 13). Table 2 lists a subset of these transcripts where at least 97% of the variance in gene expression was dependent upon mtDNA status.

**Table 2:** Transcripts where 97% of the variance in gene expression is explained by growth conditions

	Affymetrix Probe ID^a^	Gene Symbol	Entrez GeneID	Gene Description	Growth Prop. of Variance^b^	F test^c^	ρ^0 ^Vivo/ρ^0 ^Vitro		WT Vivo/WT Vivo	
							FC^d^	*P*^e^	FC^d^	*P*^e^
**Up-regulated and dependent on growth conditions**	211571_s_at	*CSPG2*	1462	chondroitin sulfate proteoglycan 2	0.99	1.41 × 10^-12^	34.6	0.0001	19.0	0.0002
	215646_s_at	*CSPG2*	1462	chondroitin sulfate proteoglycan 2	0.99	2.52 × 10^-12^	33.5	0.0003	20.3	0.0003
	220952_s_at	*PLEKHA5*	54477	PI 3-phosphate-binding protein-2	0.99	1.79 × 10^-11^	6.7	0.0003	5.6	0.0006
	209090_s_at	*SH3GLB1*	51100	SH3-domain GRB2-like endophilin B1	0.99	2.86 × 10^-10^	3.0	0.0015	3.1	0.0002
	216405_at	*LGALS1*	3956	lectin, galactoside-binding, soluble, 1	0.98	6.43 × 10^-12^	11.5	0.0007	14.4	0.0006
	212254_s_at	*DST*	667	dystonin	0.98	3.64 × 10^-11^	3.9	0.0006	4.7	0.0003
	201056_at	*GOLGB1*	2804	golgin B1, golgi integral mem. protein	0.98	2.12 × 10^-10^	3.3	0.0008	2.9	0.0004
	221829_s_at	*TNPO1*	3842	transportin 1	0.98	2.86 × 10^-10^	4.0	0.0012	3.9	0.0005
	204619_s_at	*CSPG2*	1462	chondroitin sulfate proteoglycan 2	0.98	3.01 × 10^-11^	12.2	0.0003	7.5	0.0003
	205173_x_at	*CD58*	965	CD58 molecule	0.98	1.70 × 10^-10^	3.5	0.0014	3.7	0.0004
	218901_at	*PLSCR4*	57088	phospholipid scramblase 4	0.98	1.32 × 10^-9^	3.5	0.0009	3.8	0.0014
	213229_at	*DICER1*	23405	Dicer1, Dcr-1 homolog	0.97	2.18 × 10^-10^	6.1	0.0009	7.0	0.0013
	208772_at	*ANKHD1*	404734	ankyrin repeat & KH domain containing 1	0.97	1.97 × 10^-9^	2.5	0.0027	2.4	0.0003
	221731_x_at	*CSPG2*	1462	chondroitin sulfate proteoglycan 2	0.97	4.35 × 10^-11^	104.7	0.0001	30.9	0.0002
	201057_s_at	*GOLGB1*	2804	golgin B1, golgi integral mem. protein	0.97	1.39 × 10^-9^	3.2	0.0027	2.9	0.0002
	214295_at	*KIAA0485*	57235	KIAA0485 protein	0.97	4.14 × 10^-10^	3.9	0.0014	4.1	0.0008
	201024_x_at	*EIF5B*	9669	eukaryotic translation initiation factor 5B	0.97	2.66 × 10^-9^	2.3	0.0012	2.2	0.0012
	201280_s_at	*DAB2*	1601	disabled homolog 2	0.97	4.14 × 10^-10^	5.3	0.0004	3.5	0.0006
	218396_at	*VPS13C*	54832	vacuolar protein sorting 13 homolog C	0.97	8.54 × 10^-10^	4.4	0.0012	4.8	0.0006
	205383_s_at	*ZBTB20*	26137	zinc finger and BTB domain containing 20	0.97	5.52 × 10^-10^	4.2	0.0011	6.7	0.0003
	219221_at	*ZBTB38*	253461	zinc finger and BTB domain containing 38	0.97	2.98 × 10^-12^	6.0	0.0007	6.5	0.0003
	208663_s_at	*TTC3*	7267	tetratricopeptide repeat domain 3	0.97	1.19 × 10^-9^	3.1	0.0021	3.1	0.0013
	212070_at	*GPR56*	9289	G protein-coupled receptor 56	0.97	3.78 × 10^-10^	3.3	0.0007	4.3	0.0008
	203216_s_at	*MYO6*	4646	myosin VI	0.97	2.35 × 10^-9^	3.1	0.0018	2.7	0.0012
	204620_s_at	*CSPG2*	1462	chondroitin sulfate proteoglycan 2	0.97	6.97 × 10^-11^	104.5	0.0001	27.8	0.0002
	212062_at	*ATP9A*	10079	ATPase, Class II, type 9A	0.97	2.73 × 10^-9^	2.5	0.0002	2.7	0.0030
	213775_x_at	*ZNF638*	27332	zinc finger protein 638	0.97	2.86 × 10^-10^	2.6	0.0008	3.0	0.0005
**Down-regulated and dependent on growth conditions**	213201_s_at	*TNNT1*	7138	troponin T type 1	0.99	6.43 × 10^-12^	-6.9	0.0002	-5.0	0.0003
	212432_at	*GRPEL1*	80273	GrpE-like 1, mitochondrial	0.99	1.37 × 10^-9^	-2.3	0.0008	-2.3	0.0005
	209507_at	*RPA3*	6119	replication protein A3, 14 kDa	0.98	6.76 × 10^-10^	-2.2	0.0010	-2.3	0.0002
	209825_s_at	*UCK2*	7371	uridine-cytidine kinase 2	0.98	1.09 × 10^-9^	-2.5	0.0019	-2.3	0.0006
	202533_s_at	*DHFR*	1719	dihydrofolate reductase	0.98	3.51 × 10^-9^	-2.1	0.0013	-2.0	0.0006
	201577_at	*NME1*	4830	non-metastatic cells 1, protein expressed	0.97	2.29 × 10^-9^	-2.1	0.0014	-2.0	0.0004
	208756_at	*EIF3S2*	8668	euk. transl. init. factor 3, subunit 2 beta	0.97	1.92 × 10^-8^	-1.8	0.0002	-1.7	0.0013
	207239_s_at	*PCTK1*	5127	PCTAIRE protein kinase 1	0.97	6.29 × 10^-9^	-1.9	0.0017	-1.9	0.0007
	204126_s_at	*CDC45L*	8318	CDC45 cell division cycle 45-like	0.97	2.31 × 10^-8^	-2.0	0.0030	-1.9	0.0004
	219162_s_at	MRPL11*	65003	mitochondrial ribosomal protein L11	0.97	1.37 × 10^-9^	-2.5	0.0010	-2.2	0.0006
	208847_s_at	*ADH5*	128	alcohol dehydrogenase 5, chi polypeptide	0.97	5.92 × 10^-8^	-1.7	0.0022	-1.6	0.0007
	210250_x_at	*ADSL*	158	adenylosuccinate lyase	0.97	4.04 × 10^-9^	-2.0	0.0015	-2.3	0.0012
	208799_at	*PSMB5*	5693	proteasome beta 5 subunit	0.97	2.6 × 10^-8^	-1.9	0.0025	-1.8	0.0004
	221620_s_at	*NOMO3*	408050	NODAL modulator 3	0.97	1.64 × 10^-9^	-2.0	0.0015	-2.3	0.0004
	210519_s_at	*NQO1*	1728	NAD(P)H dehydrogenase, quinone 1	0.97	7.60 × 10^-9^	-2.1	0.0020	-1.8	0.0007
	208910_s_at	*C1QBP*	708	splicing factor SF2-associated protein	0.97	1.63 × 10^-9^	-2.4	0.0020	-2.5	0.0008
	201903_at	*UQCRC1*	7384	ubiquinol-cytochrome c reductase	0.97	9.25 × 10^-9^	-1.8	0.0019	-2.1	0.0009
	200039_s_at	*PSMB2*	5690	proteasome subunit, beta type, 2	0.97	5.72 × 10^-9^	-1.9	0.0022	-2.0	0.0006
	217960_s_at	*TOMM22*	56993	mitochondrial import receptor Tom22	0.97	1.04 × 10^-8^	-2.2	0.0007	-1.9	0.0014
	205691_at	*SYNGR3*	9143	synaptogyrin 3	0.97	1.83 × 10^-9^	-3.1	0.0012	-2.4	0.0012

^a^Only probe tilings representing genes with annotated functions are provided

^b^Proportion of variance in gene expression due to growth conditions (i.e. cultured cells or xenografts)

^c^Bayes modified F test (multiple hypothesis corrected)

^d^Fold-change ^e^Benjamini and Hochberg corrected two-tailed Student's t-test

Of the GO categories enriched for transcripts whose expression was highly dependent upon growth conditions, only the nuclear RNA splicing GO category (9 transcripts) were more abundant in xenografts relative to cultured cells (Additional File 14A). However, multiple GO categories were enriched for transcripts whose variance was principally explained by growth conditions and were less abundant in xenografts relative to cell culture. These included categories related to mitochondrial function (50 mitochondrion transcripts), glycolysis (7 transcripts), electron carrier activity (11 transcripts), purine metabolism (5 transcripts), and the proteosome core complex (6 transcripts) (Additional File 14B).

The decrease in mitochondrial transcripts encoded by the nuclear genome may relate to the relative oxygen deprivation in xenografts relative to cell culture which would cause cells to rely less upon oxidative phosphorylation for their energy needs. The fact that growth conditions play a larger role in the expression of nuclear-encoded mitochondrial genes than the presence of the mitochondrial genome is not surprising if one considers the fact that ρ^0 ^cells form mitochondrial structures in the absence of mtDNA. Furthermore, this is consistent with observations that there are no differences in the expression of nuclear-encoded oxidative phosphorylation genes in parental and mtDNA-deficient osteosarcoma cells [71]. Interestingly, we could not detect differential transcript levels of nuclear encoded mtDNA polymerase (*POLG*), previously reported to be decreased on the protein level in HeLa ρ^0 ^cells [72], in any pair-wise comparison of experimental grouping performed in this study. However, we did find growth conditions accounted for 73% of the variance in gene expression (corrected F test *P *= 8.3 × 10^-6 ^and down-regulated in xenografts) of the nuclear encoded mitochondrial RNA polymerase *POLRMT*, which was reported to be down-regulated on the protein level in response to mtDNA depletion in HeLa cells [72].

The above observations, also supported by KEGG (Kyoto Encyclopedia of Genes and Genomes) pathway analyses of the same group of transcripts (Additional File 15), indicate that both glycolysis and aerobic respiration-related genes are less abundant in xenografts relative to culture. This could relate to the cells being cultured in glucose-rich medium that does not reflect the situation found *in vivo*. In combination, decreased glycolysis and oxidative phosphorylation in xenografts reflects lower energetic demands *in vivo*. Likewise, the decreased abundance of transcripts related to purine metabolism in xenografts is likely related to slower growth rates in xenografts relative to culture. Decreased purine metabolism could also reflect the absence of the super-physiological levels of glutamine present in cell culture media.

### Role of mtDNA mutations in human disorders

A549 lung cancer cells provide an appealing model system to study mitochondrial function due to their ability to be grown in culture and in tumor xenografts. The potential role of mitochondrial function in cancer was first recognized by Otto Warburg when he observed that cancer cells show a characteristic shift in energy production from oxidative phosphorylation to glycolysis [73]. More recently, there has been increasing appreciation of the possible importance of inherited and somatic mtDNA mutations in cancer [74]. Based on the gene expression profiles observed in this study, ρ^0 ^xenograft tumor models could be of particular interest in determining mtDNA-mediated responses to specific cancer therapies, such anti-angiogenic agents.

In addition, ρ^0 ^cells play a critical role in the production of cybrid (cytoplasmic hybrid) cell lines containing specific mutations in mtDNA. While multiple groups have used this elegant system to study mitochondrial function in cultured cells (see reviews [4,75]), only recently have these cybrids been used in tumor xenografts [64]. Gene expression profiling of such cybrids both in culture and in xenografts could provide valuable data to elucidate further the functional interactions between the nuclear and mitochondrial genomes.

## Conclusion

Although growth conditions had a greater influence on gene expression profiles than the presence of active mitochondria, we identified mtDNA-dependent gene expression profiles that are shared in cultured A549 cells and in xenografts. These profiles indicate that cells with mtDNA alterations have distinct biochemical properties that make them well-suited for elucidating responses to physiological perturbations, such as changes in oxygen and glucose levels, and testing the effects of specialized classes of chemotherapeutic agents. In addition, our studies suggest that gene expression profiling of mtDNA-depleted cells grown culture and in xenografts provide powerful means to investigate possible relationships between mitochondrial activity and gene expression profiles in normal and pathological cells.

## Authors' contributions

JP carried out flow cytometry and immunoassays. PT and XM performed the xenograft studies. PL prepared mtDNA-depleted cells, performed oxygen consumption, flow cytometry, and ELISA assays, and participated in the design of the study. PKD performed gene expression profiling experiments and assisted in data analysis. KR performed mtDNA quantification studies. DMT and KDS analyzed gene expression data. DM and JH conceived of the study, interpreted the data, and wrote the manuscript.

## Supplementary Material

Additional file 1 **Analysis of mitochondrial-encoded RNA transcript levels in A549 and A549 ρ0 xenografts by quantitative RT-PCR.** Mitochondrial-encoded RNA transcript levels in A549 and A549 ρ0 xenografts are quantified by RT-PCR. Click here for file

Additional file 2 **Processed gene expression scores for all microarray analyses.** Gene expression scores for all microarray experiments described in this manuscript. Click here for file

Additional file 3 **Volcano plots for gene expression comparisons considered in this study.** The relationships between fold changes and P-values for the gene expression comparisons made in this study are provided. Click here for file

Additional file 4 **Differentially expressed transcripts in A549 ρ^0 ^and A549 cells grown in culture. **Probe sets showing differential expression in cultured A549 ρ^0 ^and A549 cells are provided. Click here for file

Additional file 5 **Gene Ontology analysis of transcripts that are differentially expressed in A549 ρ^0 ^cells relative to parental A549 cells in culture.** Functional categories of transcripts showing differential expression in cultured A549 ρ^0 ^and A549 cells are provided. Click here for file

Additional file 6 **Analysis of HIF-1 responsive genes in A549 ρ^0 ^and A549 cells.** Gene expression profiles of HIF-1 responsive genes in A549 ρ^0 ^and A549 cells are provided. Click here for file

Additional file 7 **Transcripts that are differentially expressed in A549 ρ^0 ^and A549 cells grown in xenografts.** Probe sets showing differential expression in A549 ρ^0 ^and A549 xenografts are provided. Click here for file

Additional file 8 **Gene Ontology analysis of transcripts that are differentially expressed in A549 ρ^0 ^cells relative to parental A549 cells in xenografts.** Functional categories of transcripts showing differential expression in cultured A549 ρ^0 ^and A549 xenografts are provided. Click here for file

Additional file 9 **Differential expression in A549 xenografts and A549 cell cultures.** Probe sets showing differential expression in A549 xenografts and A549 cell cultures are provided. Click here for file

Additional file 10 **Differential expression in A549 ρ^0 ^xenografts and A549 ρ^0 ^cell cultures.** Probe sets showing differential expression in A549 ρ^0^xenografts and A549 ρ^0 ^cell cultures are provided. Click here for file

Additional file 11 **Probe sets where 50% or more of the variance in gene expression profiles is influenced by mtDNA status. **Probe sets where 50% or more of the variance in gene expression profiles is influenced by mtDNA status are provided. Click here for file

Additional file 12 **Gene Ontology analysis of down-regulated transcripts in ρ^0 ^cells whose variance is strongly influenced by mtDNA status. **Functional categories of down-regulated transcripts in ρ^0 ^cells whose variance is strongly influenced by mtDNA status are provided. Click here for file

Additional file 13 **Probe sets where 90% or more of the variance in gene expression profiles is influenced by growth conditions. **Probe sets where 90% or more of the variance in gene expression profiles is influenced by growth conditions are provided. Click here for file

Additional file 14 **Gene Ontology analysis of transcripts whose variance are strongly dependent upon growth conditions. **Functional categories of transcripts whose variance are strongly dependent upon growth conditions are provided. Click here for file

Additional file 15 **KEGG analysis of differentially expressed genes in A549 ρ^0 ^and A549 cells. **Biological pathways containing differentially expressed genes in A549 ρ^0 ^and A549 cells are provided. Click here for file
