# Supplementary material for: mtDNA depletion confers specific gene expression profiles in human cells grown in culture and in xenograft
Source: BMC Genomics. 2008 Nov 3;9:521. doi: 10.1186/1471-2164-9-521 (PMC2612029; doi:10.1186/1471-2164-9-521)
Supplement: Additional file 1 — Analysis of mitochondrial-encoded RNA transcript levels in A549 and A549 ρ0 xenografts by quantitative RT-PCR. Mitochondrial-encoded RNA transcript levels in A549 and A549 ρ0 xenografts are quantified by RT-PCR. [file 1471-2164-9-521-S1.ppt]

## Slide 1
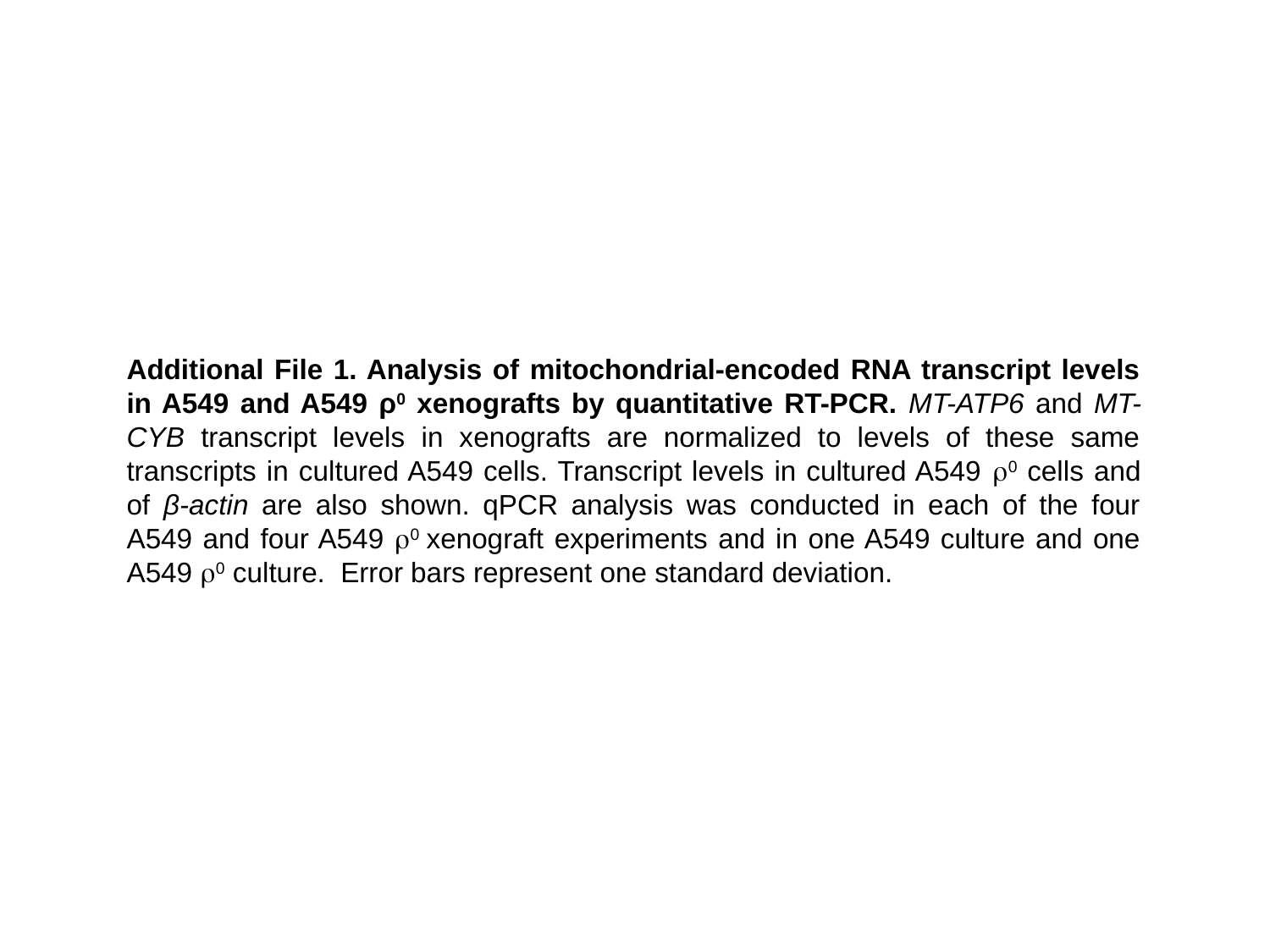

Additional File 1. Analysis of mitochondrial-encoded RNA transcript levels in A549 and A549 ρ0 xenografts by quantitative RT-PCR. MT-ATP6 and MT-CYB transcript levels in xenografts are normalized to levels of these same transcripts in cultured A549 cells. Transcript levels in cultured A549 0 cells and of β-actin are also shown. qPCR analysis was conducted in each of the four A549 and four A549 0 xenograft experiments and in one A549 culture and one A549 0 culture. Error bars represent one standard deviation.

## Slide 2
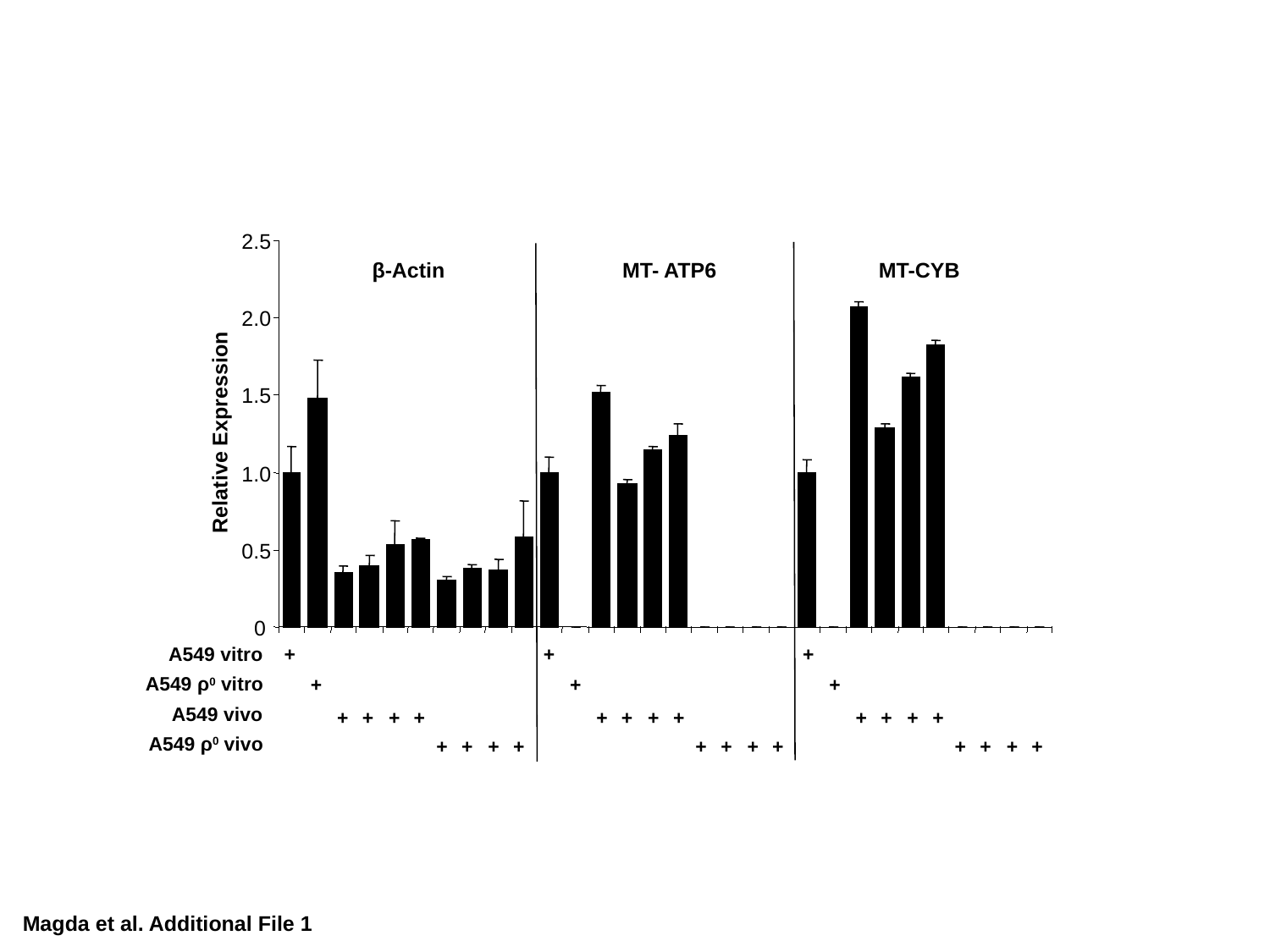

2.5
β-Actin
MT- ATP6
MT-CYB
2.0
1.5
Relative Expression
1.0
0.5
0
A549 vitro
+
+
+
+
+
+
+
+
+
+
+
+
+
+
+
+
+
+
+
+
+
+
+
+
+
+
+
+
+
+
A549 ρ0 vitro
A549 vivo
A549 ρ0 vivo
Magda et al. Additional File 1
