# Supplementary material for: mtDNA depletion confers specific gene expression profiles in human cells grown in culture and in xenograft
Source: BMC Genomics. 2008 Nov 3;9:521. doi: 10.1186/1471-2164-9-521 (PMC2612029; doi:10.1186/1471-2164-9-521)
Supplement: Additional file 3 — Volcano plots for gene expression comparisons considered in this study. The relationships between fold changes and P-values for the gene expression comparisons made in this study are provided. [file 1471-2164-9-521-S3.ppt]

## Slide 1
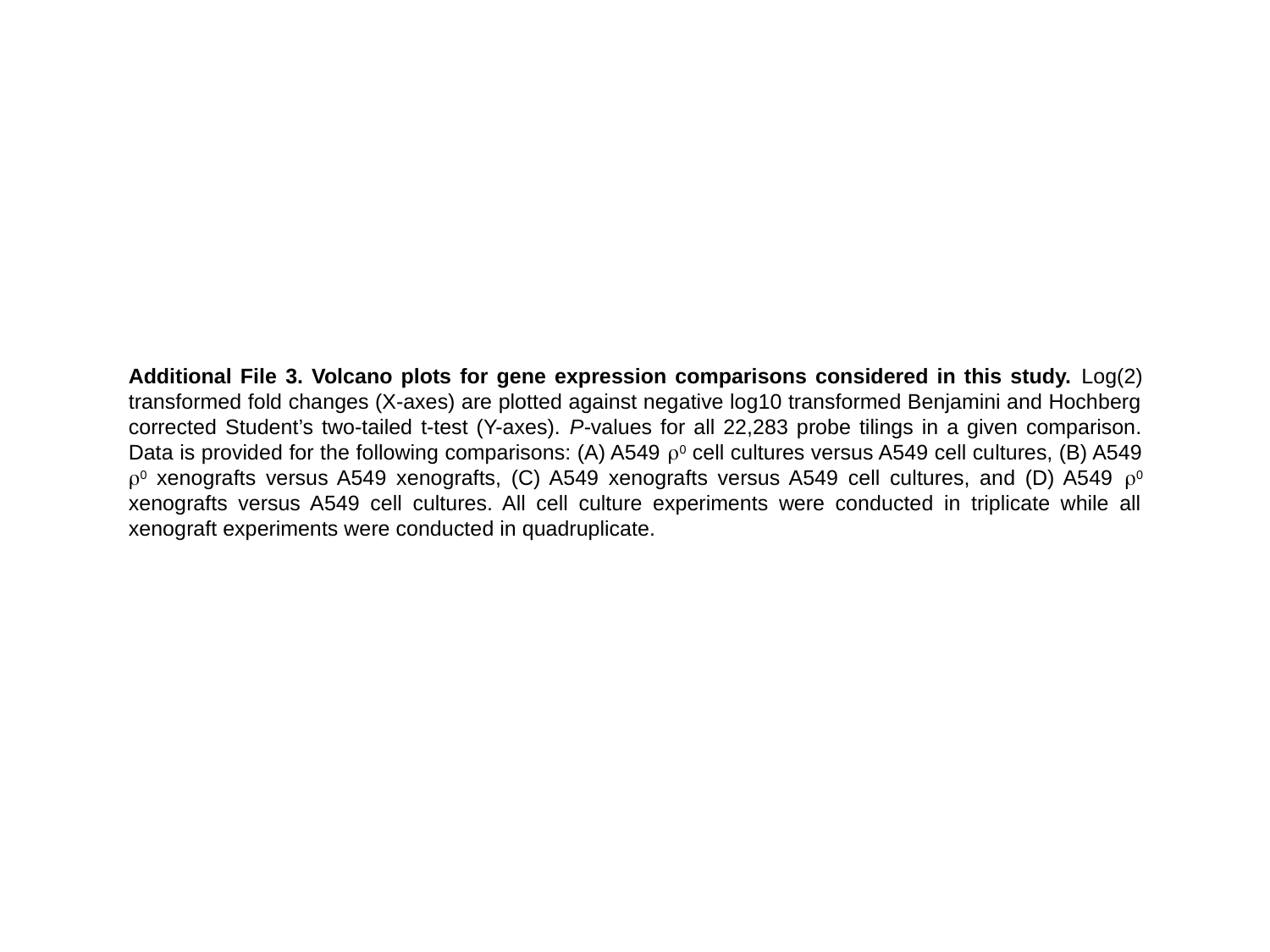

Additional File 3. Volcano plots for gene expression comparisons considered in this study. Log(2) transformed fold changes (X-axes) are plotted against negative log10 transformed Benjamini and Hochberg corrected Student’s two-tailed t-test (Y-axes). P-values for all 22,283 probe tilings in a given comparison. Data is provided for the following comparisons: (A) A549 0 cell cultures versus A549 cell cultures, (B) A549 0 xenografts versus A549 xenografts, (C) A549 xenografts versus A549 cell cultures, and (D) A549 0 xenografts versus A549 cell cultures. All cell culture experiments were conducted in triplicate while all xenograft experiments were conducted in quadruplicate.

## Slide 2
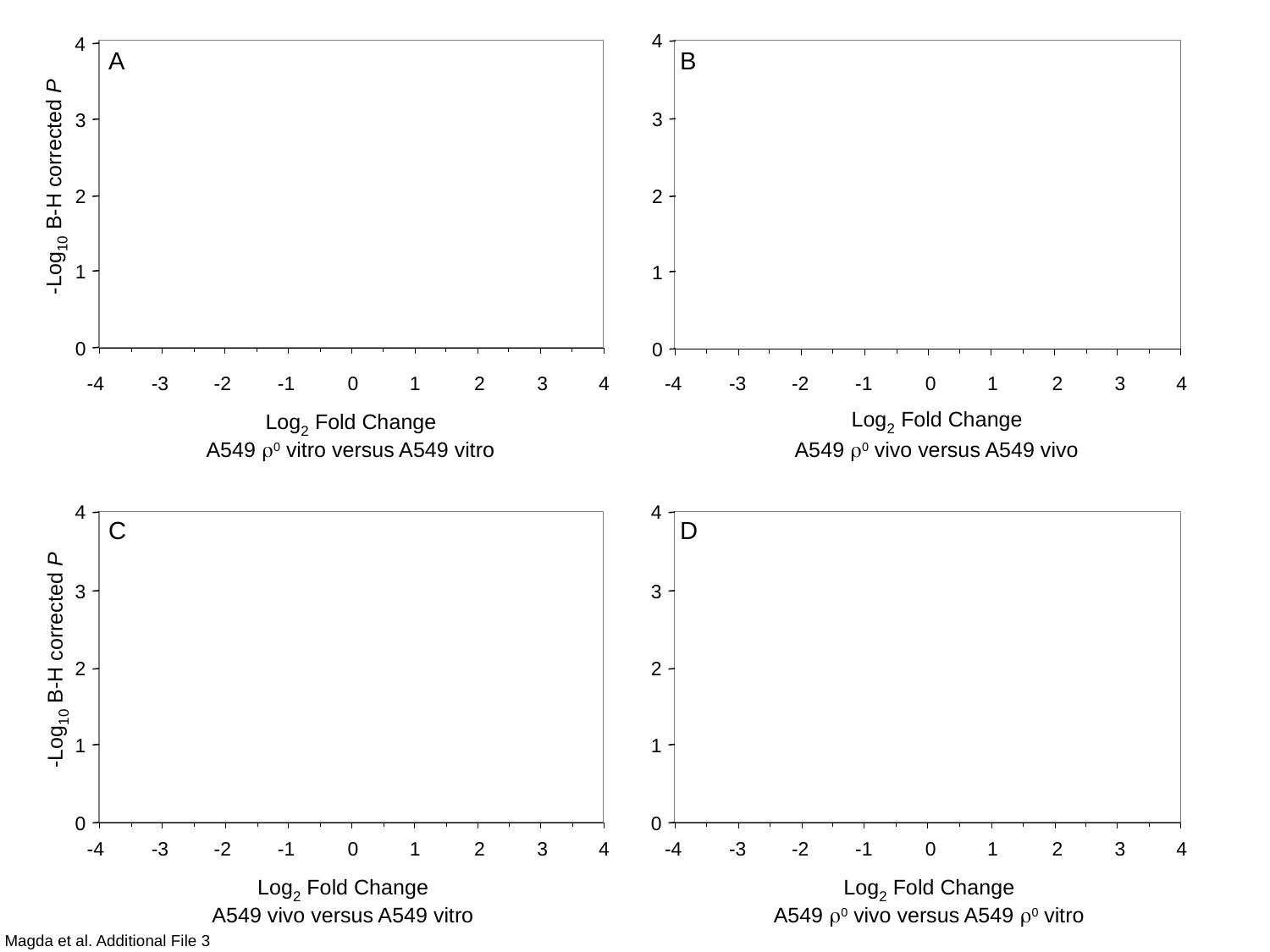

4
3
2
1
0
4
A
B
3
-Log10 B-H corrected P
2
1
0
-4
-3
-2
-1
0
1
2
3
4
-4
-3
-2
-1
0
1
2
3
4
Log2 Fold Change
Log2 Fold Change
A549 0 vitro versus A549 vitro
A549 0 vivo versus A549 vivo
4
3
2
1
0
4
3
2
1
0
C
D
-Log10 B-H corrected P
-4
-3
-2
-1
0
1
2
3
4
-4
-3
-2
-1
0
1
2
3
4
Log2 Fold Change
Log2 Fold Change
A549 vivo versus A549 vitro
A549 0 vivo versus A549 0 vitro
Magda et al. Additional File 3
