# Supplementary material for: mtDNA depletion confers specific gene expression profiles in human cells grown in culture and in xenograft
Source: BMC Genomics. 2008 Nov 3;9:521. doi: 10.1186/1471-2164-9-521 (PMC2612029; doi:10.1186/1471-2164-9-521)
Supplement: Additional file 5 — Gene Ontology analysis of transcripts that are differentially expressed in A549 ρ0 cells relative to parental A549 cells in culture. Functional categories of transcripts showing differential expression in cultured A549 ρ0 and A549 cells are provided. [file 1471-2164-9-521-S5.ppt]

## Slide 1
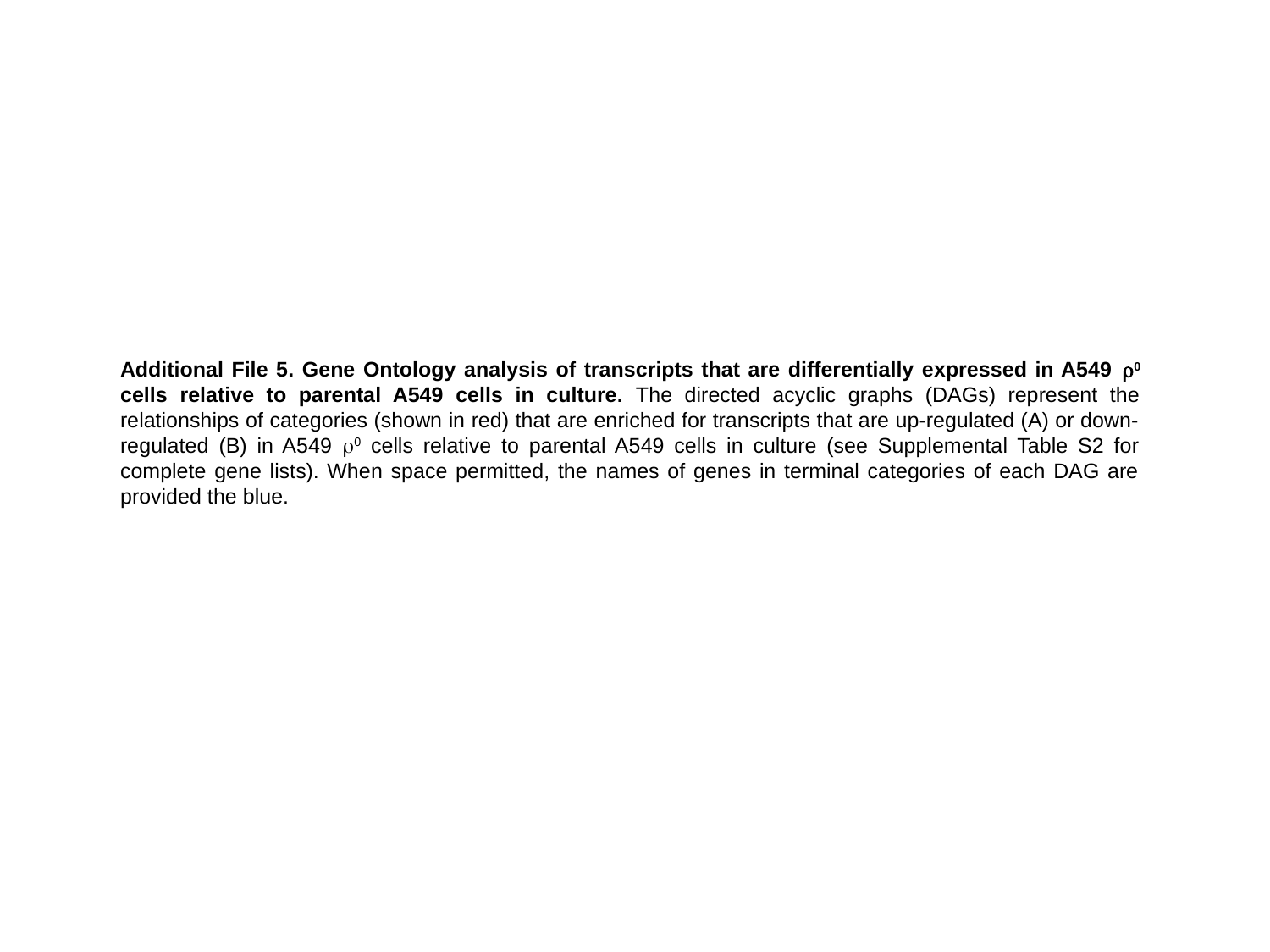

Additional File 5. Gene Ontology analysis of transcripts that are differentially expressed in A549 0 cells relative to parental A549 cells in culture. The directed acyclic graphs (DAGs) represent the relationships of categories (shown in red) that are enriched for transcripts that are up-regulated (A) or down-regulated (B) in A549 0 cells relative to parental A549 cells in culture (see Supplemental Table S2 for complete gene lists). When space permitted, the names of genes in terminal categories of each DAG are provided the blue.

## Slide 2
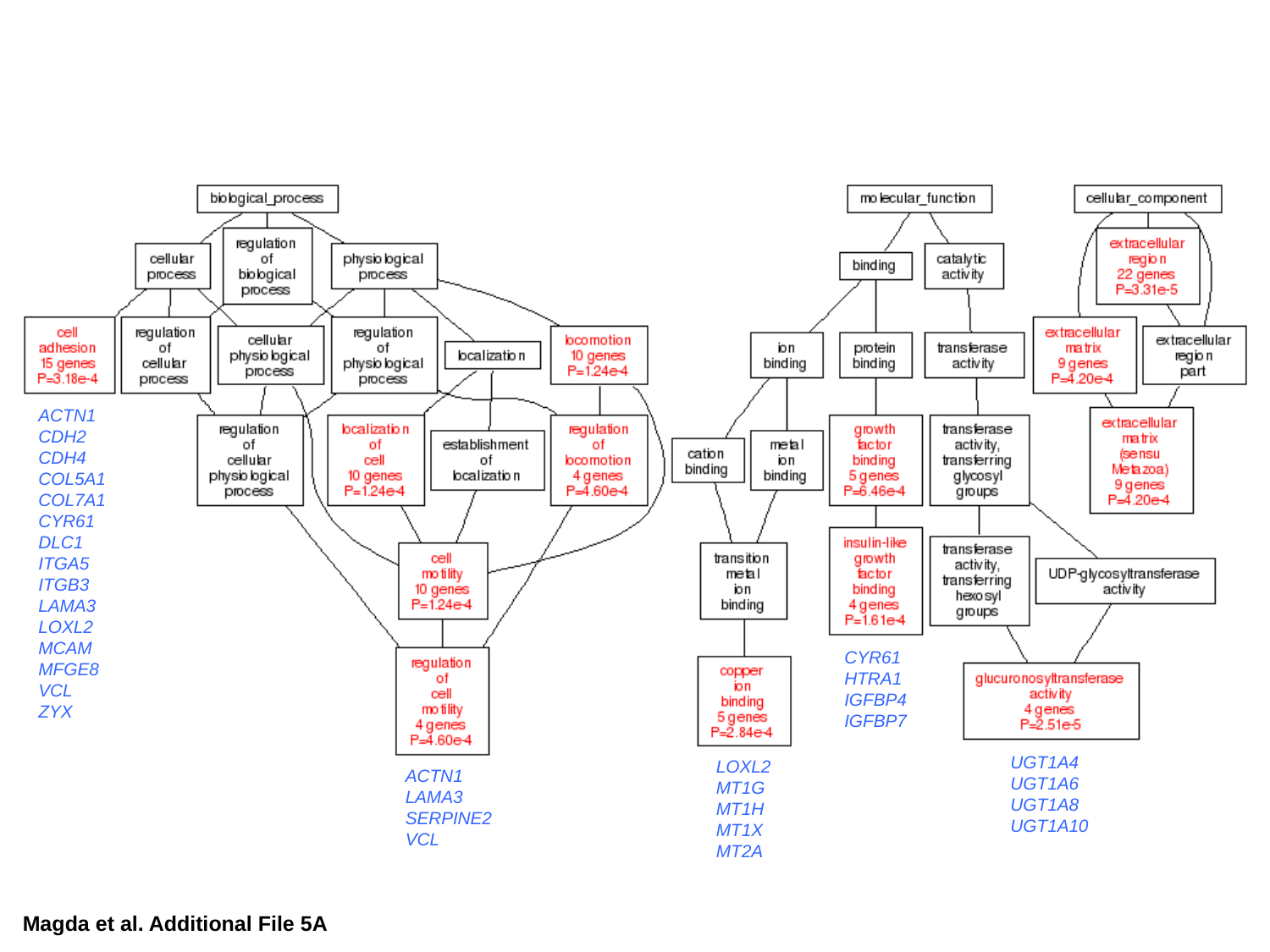

ACTN1
CDH2
CDH4
COL5A1
COL7A1
CYR61
DLC1
ITGA5
ITGB3
LAMA3
LOXL2
MCAM
MFGE8
VCL
ZYX
CYR61
HTRA1
IGFBP4
IGFBP7
UGT1A4
UGT1A6
UGT1A8
UGT1A10
LOXL2
MT1G
MT1H
MT1X
MT2A
ACTN1
LAMA3
SERPINE2
VCL
Magda et al. Additional File 5A

## Slide 3
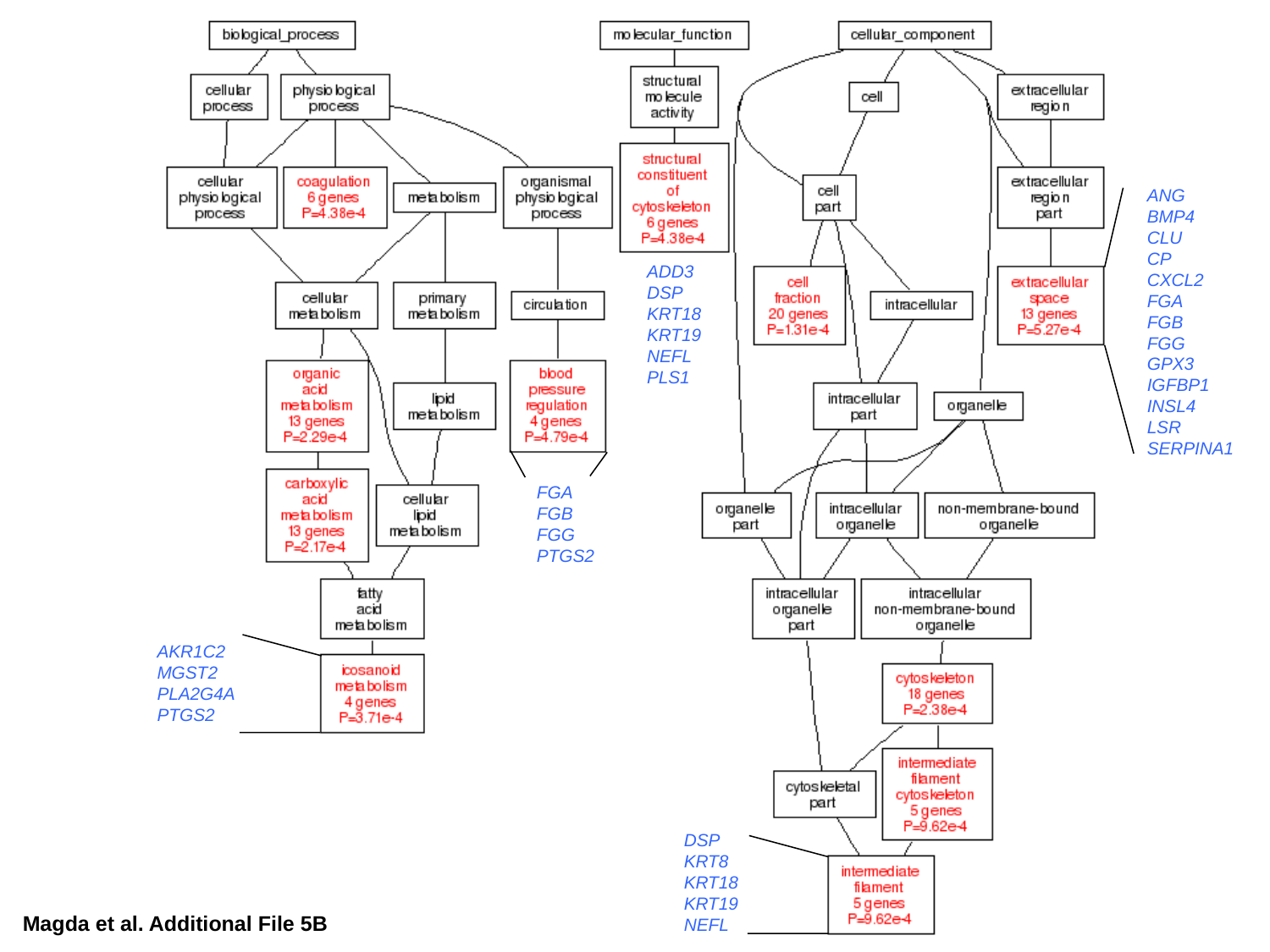

ANG
BMP4
CLU
CP
CXCL2
FGA
FGB
FGG
GPX3
IGFBP1
INSL4
LSR
SERPINA1
ADD3
DSP
KRT18
KRT19
NEFL
PLS1
FGA
FGB
FGG
PTGS2
AKR1C2
MGST2
PLA2G4A
PTGS2
DSP
KRT8
KRT18
KRT19
NEFL
Magda et al. Additional File 5B
