# Supplementary material for: mtDNA depletion confers specific gene expression profiles in human cells grown in culture and in xenograft
Source: BMC Genomics. 2008 Nov 3;9:521. doi: 10.1186/1471-2164-9-521 (PMC2612029; doi:10.1186/1471-2164-9-521)
Supplement: Additional file 8 — Gene Ontology analysis of transcripts that are differentially expressed in A549 ρ0 cells relative to parental A549 cells in xenografts. Functional categories of transcripts showing differential expression in cultured A549 ρ0 and A549 xenografts are provided. [file 1471-2164-9-521-S8.ppt]

## Slide 1
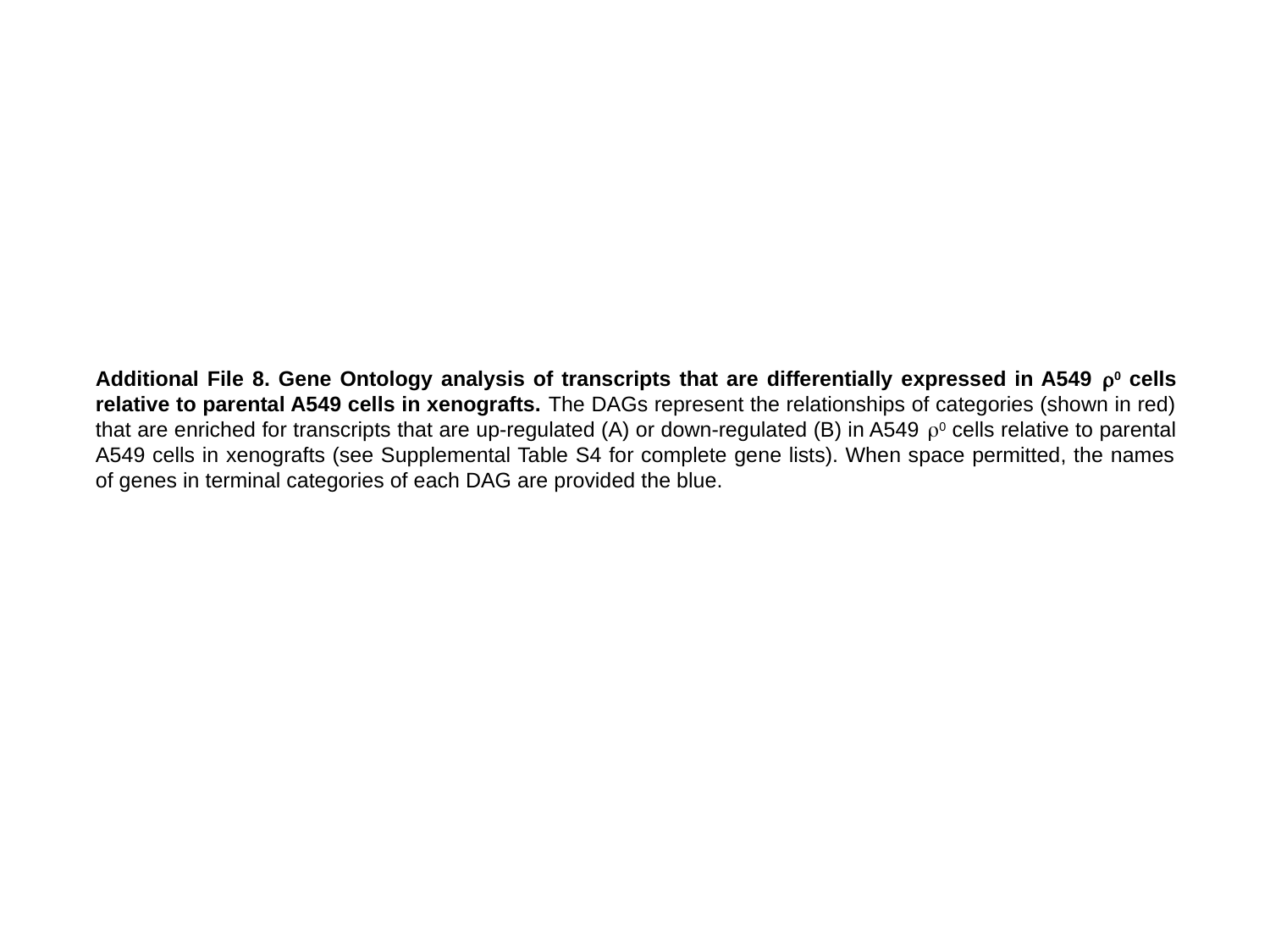

Additional File 8. Gene Ontology analysis of transcripts that are differentially expressed in A549 0 cells relative to parental A549 cells in xenografts. The DAGs represent the relationships of categories (shown in red) that are enriched for transcripts that are up-regulated (A) or down-regulated (B) in A549 0 cells relative to parental A549 cells in xenografts (see Supplemental Table S4 for complete gene lists). When space permitted, the names of genes in terminal categories of each DAG are provided the blue.

## Slide 2
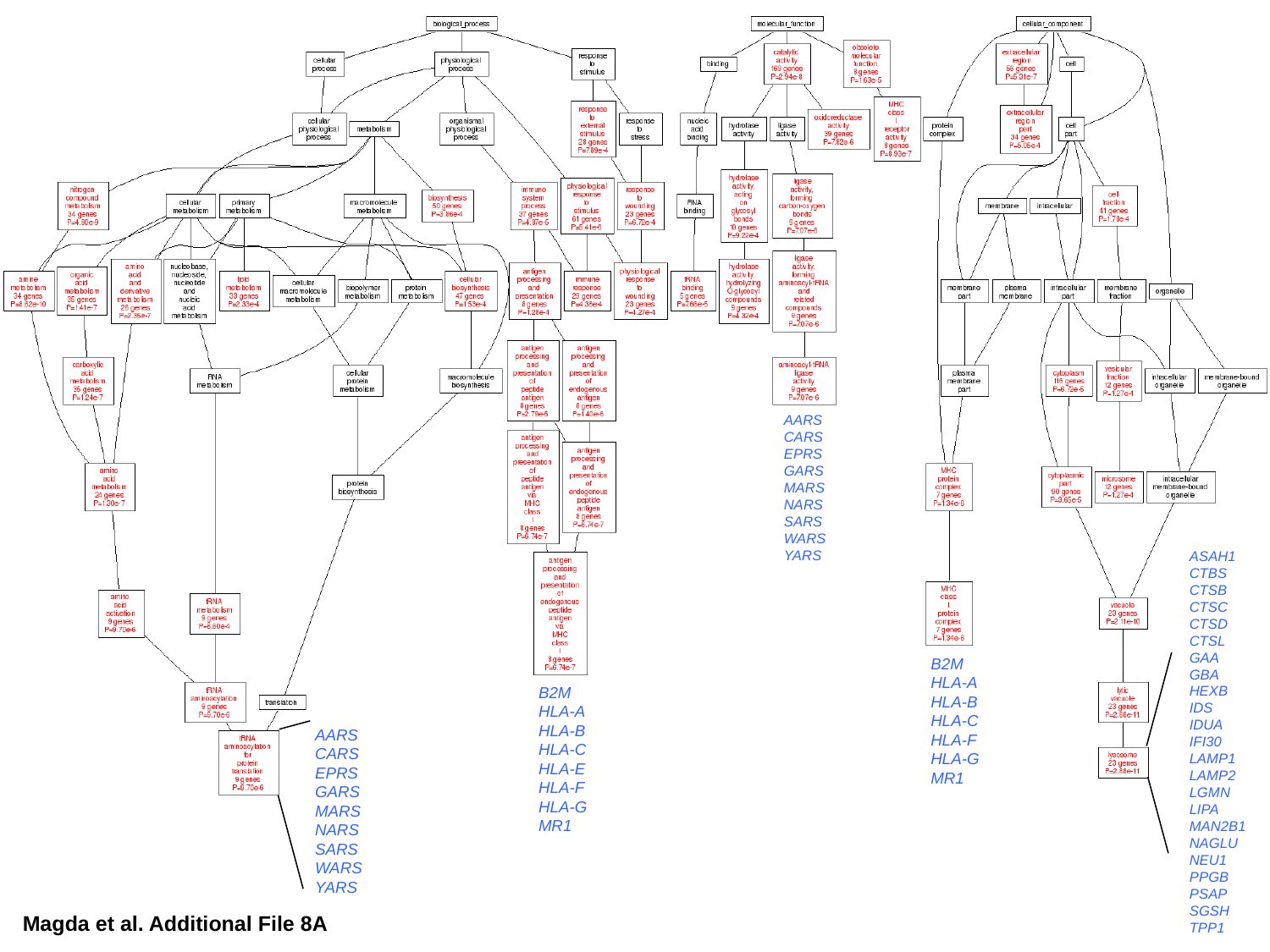

AARS
CARS
EPRS
GARS
MARS
NARS
SARS
WARS
YARS
ASAH1
CTBS
CTSB
CTSC
CTSD
CTSL
GAA
GBA
HEXB
IDS
IDUA
IFI30
LAMP1
LAMP2
LGMN
LIPA
MAN2B1
NAGLU
NEU1
PPGB
PSAP
SGSH
TPP1
B2M
HLA-A
HLA-B
HLA-C
HLA-F
HLA-G
MR1
B2M
HLA-A
HLA-B
HLA-C
HLA-E
HLA-F
HLA-G
MR1
AARS
CARS
EPRS
GARS
MARS
NARS
SARS
WARS
YARS
Magda et al. Additional File 8A

## Slide 3
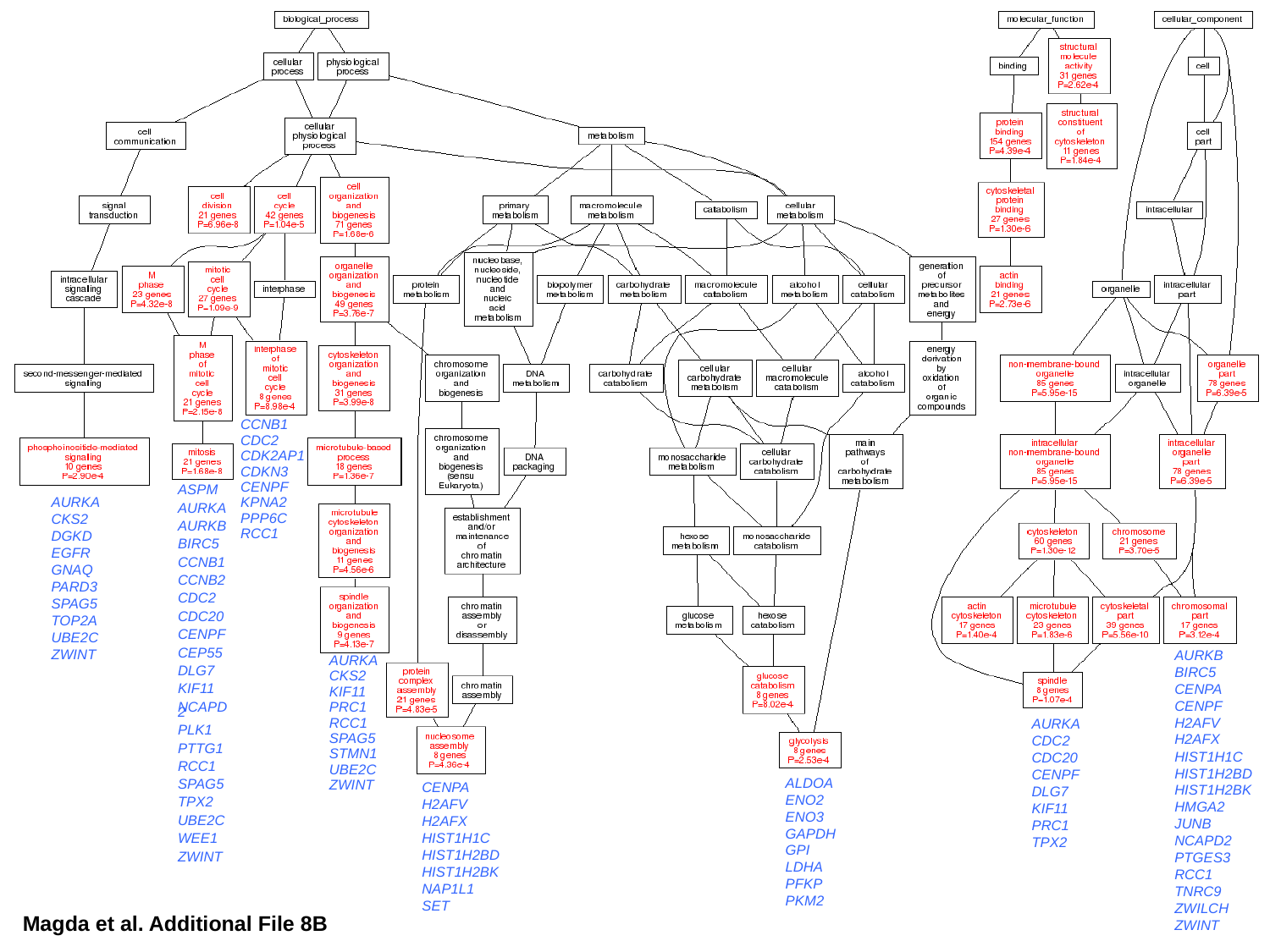

CCNB1
CDC2
CDK2AP1
CDKN3
CENPF
KPNA2
PPP6C
RCC1
| ASPM |
| --- |
| AURKA |
| AURKB |
| BIRC5 |
| CCNB1 |
| CCNB2 |
| CDC2 |
| CDC20 |
| CENPF |
| CEP55 |
| DLG7 |
| KIF11 |
| NCAPD2 |
| PLK1 |
| PTTG1 |
| RCC1 |
| SPAG5 |
| TPX2 |
| UBE2C |
| WEE1 |
| ZWINT |
AURKA
CKS2
DGKD
EGFR GNAQ
PARD3
SPAG5 TOP2A
UBE2C ZWINT
AURKB
BIRC5
CENPA
CENPF
H2AFV
H2AFX
HIST1H1C
HIST1H2BD
HIST1H2BK
HMGA2
JUNB
NCAPD2
PTGES3
RCC1
TNRC9
ZWILCH
ZWINT
AURKA
CKS2
KIF11
PRC1
RCC1
SPAG5
STMN1
UBE2C
ZWINT
AURKA
CDC2
CDC20
CENPF
DLG7
KIF11
PRC1
TPX2
ALDOA
ENO2
ENO3
GAPDH
GPI
LDHA
PFKP
PKM2
CENPA
H2AFV
H2AFX
HIST1H1C
HIST1H2BD
HIST1H2BK
NAP1L1
SET
Magda et al. Additional File 8B
