# Supplementary material for: mtDNA depletion confers specific gene expression profiles in human cells grown in culture and in xenograft
Source: BMC Genomics. 2008 Nov 3;9:521. doi: 10.1186/1471-2164-9-521 (PMC2612029; doi:10.1186/1471-2164-9-521)
Supplement: Additional file 12 — Gene Ontology analysis of down-regulated transcripts in ρ0 cells whose variance is strongly influenced by mtDNA status. Functional categories of down-regulated transcripts in ρ0 cells whose variance is strongly influenced by mtDNA status are provided. [file 1471-2164-9-521-S12.ppt]

## Slide 1
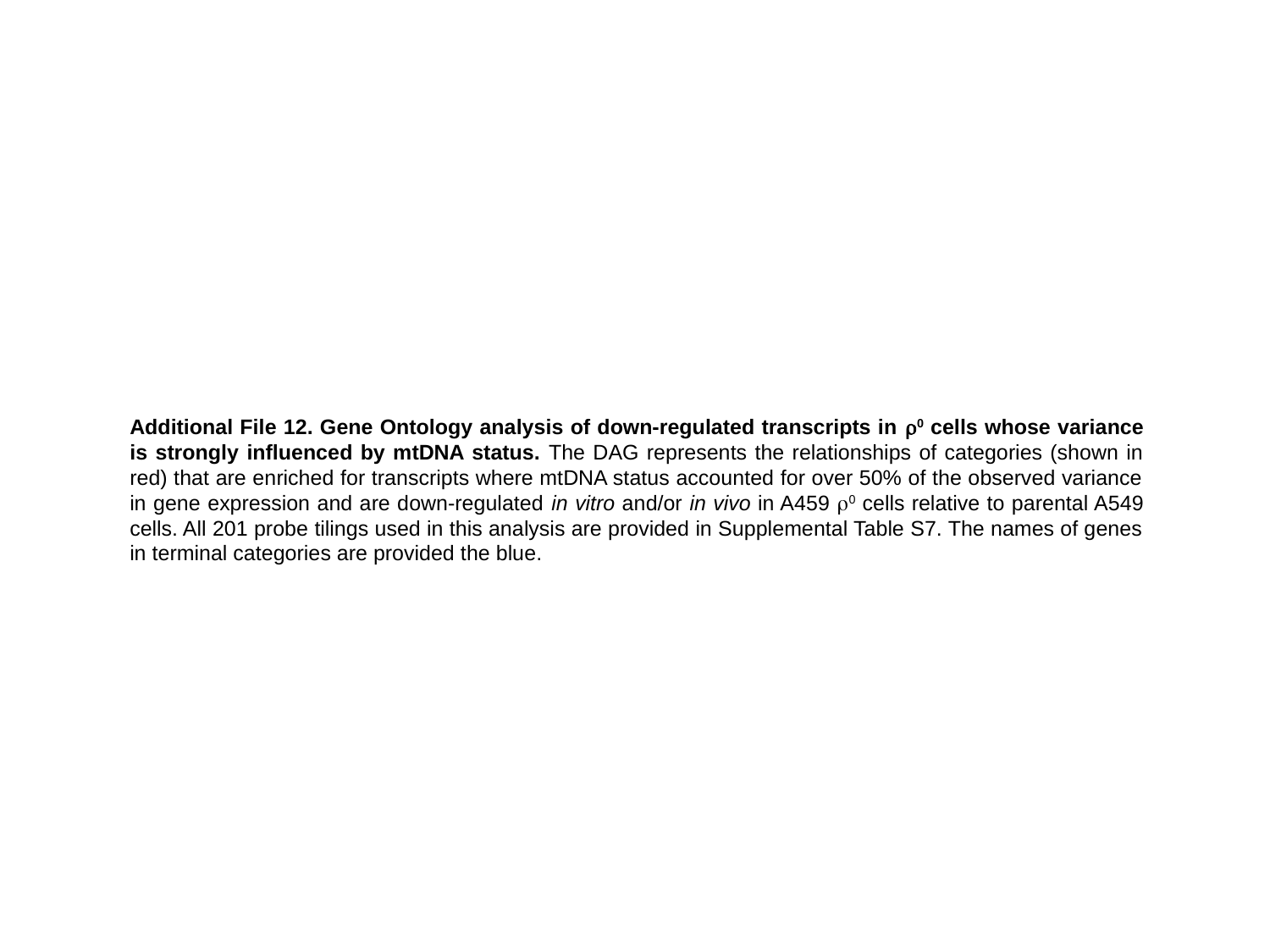

Additional File 12. Gene Ontology analysis of down-regulated transcripts in 0 cells whose variance is strongly influenced by mtDNA status. The DAG represents the relationships of categories (shown in red) that are enriched for transcripts where mtDNA status accounted for over 50% of the observed variance in gene expression and are down-regulated in vitro and/or in vivo in A459 0 cells relative to parental A549 cells. All 201 probe tilings used in this analysis are provided in Supplemental Table S7. The names of genes in terminal categories are provided the blue.

## Slide 2
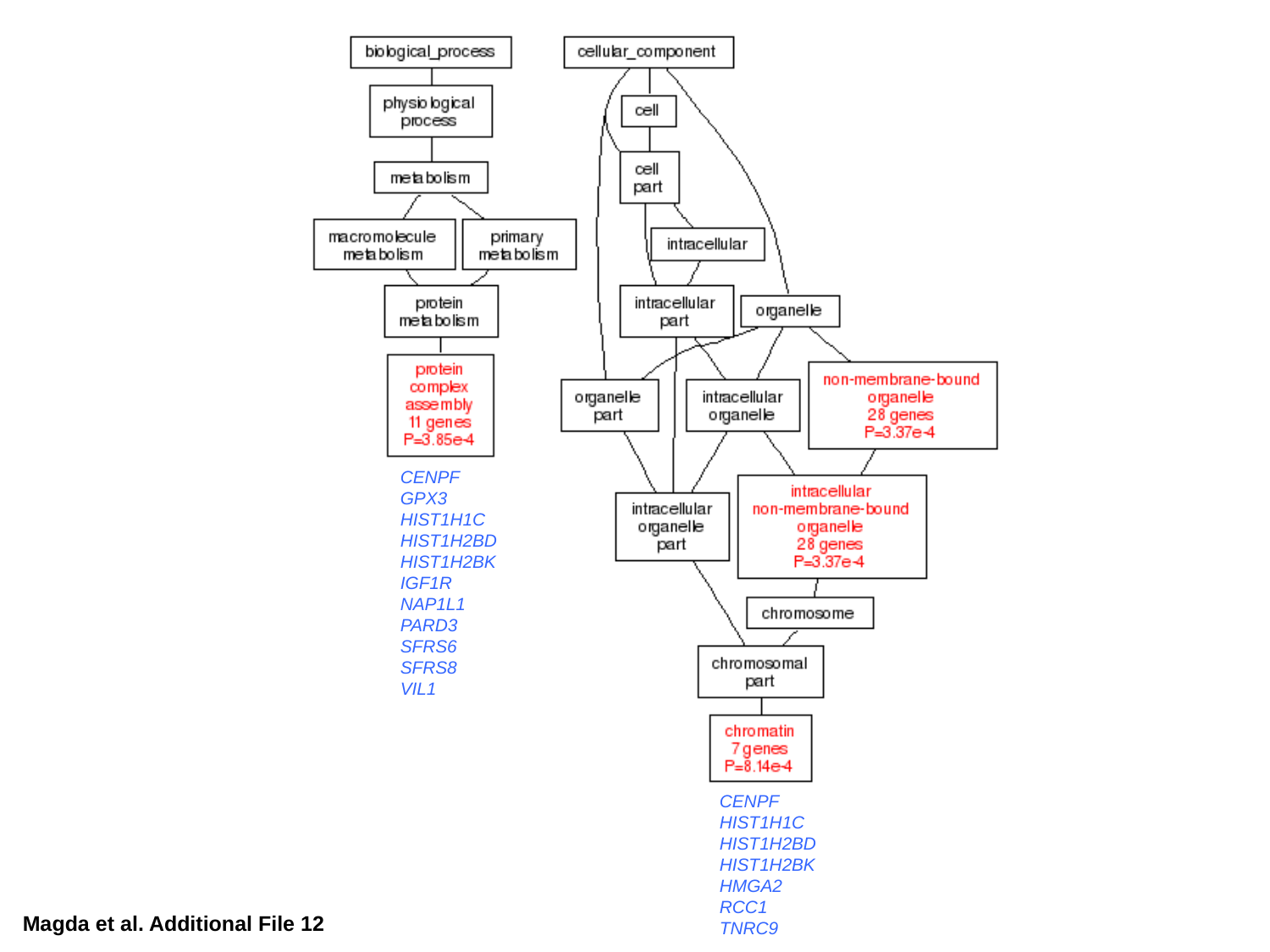

CENPF
GPX3
HIST1H1C
HIST1H2BD
HIST1H2BK
IGF1R
NAP1L1
PARD3
SFRS6
SFRS8
VIL1
CENPF
HIST1H1C
HIST1H2BD
HIST1H2BK
HMGA2
RCC1
TNRC9
Magda et al. Additional File 12
