# Supplementary material for: mtDNA depletion confers specific gene expression profiles in human cells grown in culture and in xenograft
Source: BMC Genomics. 2008 Nov 3;9:521. doi: 10.1186/1471-2164-9-521 (PMC2612029; doi:10.1186/1471-2164-9-521)
Supplement: Additional file 14 — Gene Ontology analysis of transcripts whose variance are strongly dependent upon growth conditions. Functional categories of transcripts whose variance are strongly dependent upon growth conditions are provided. [file 1471-2164-9-521-S14.ppt]

## Slide 1
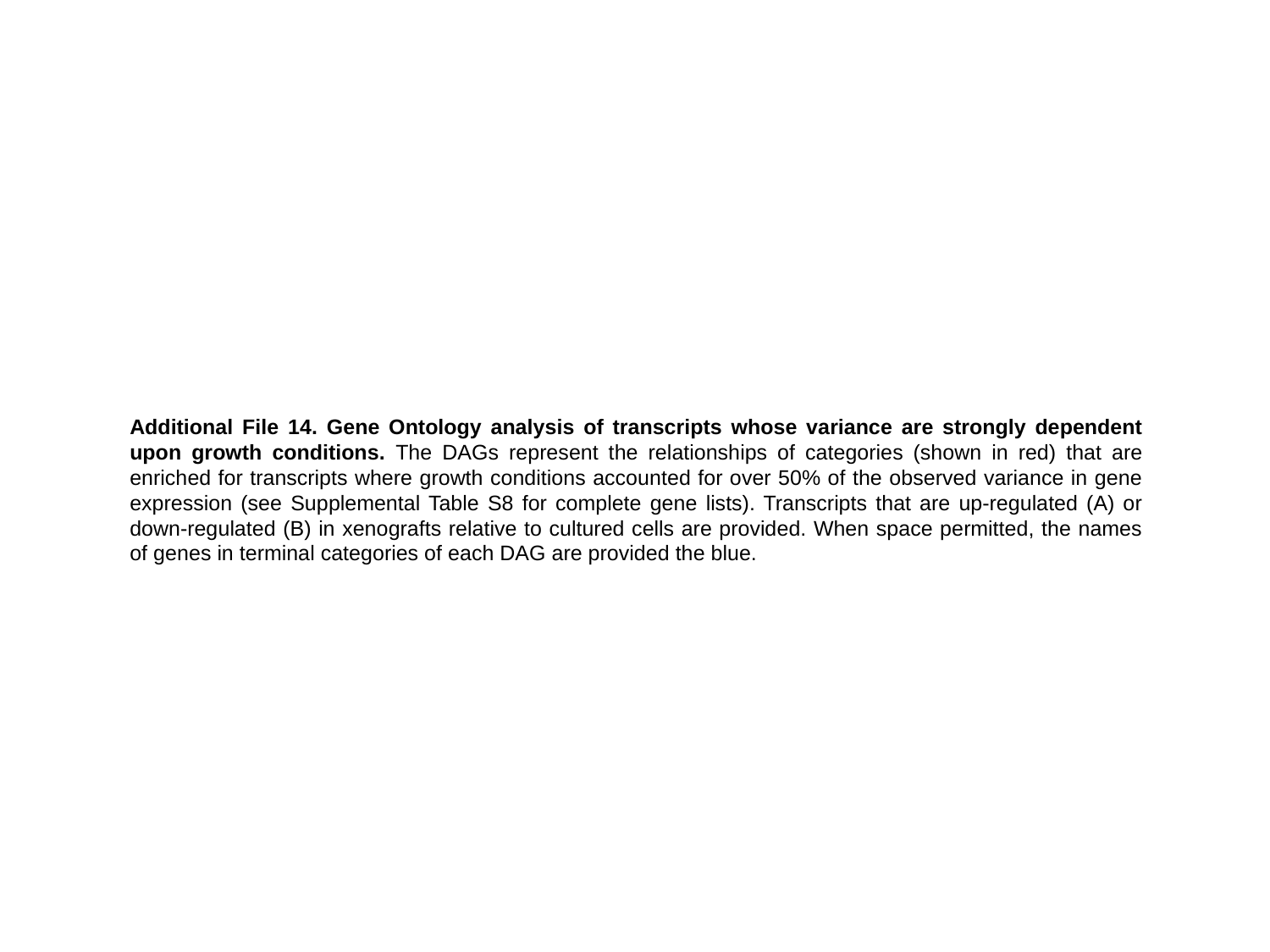

Additional File 14. Gene Ontology analysis of transcripts whose variance are strongly dependent upon growth conditions. The DAGs represent the relationships of categories (shown in red) that are enriched for transcripts where growth conditions accounted for over 50% of the observed variance in gene expression (see Supplemental Table S8 for complete gene lists). Transcripts that are up-regulated (A) or down-regulated (B) in xenografts relative to cultured cells are provided. When space permitted, the names of genes in terminal categories of each DAG are provided the blue.

## Slide 2
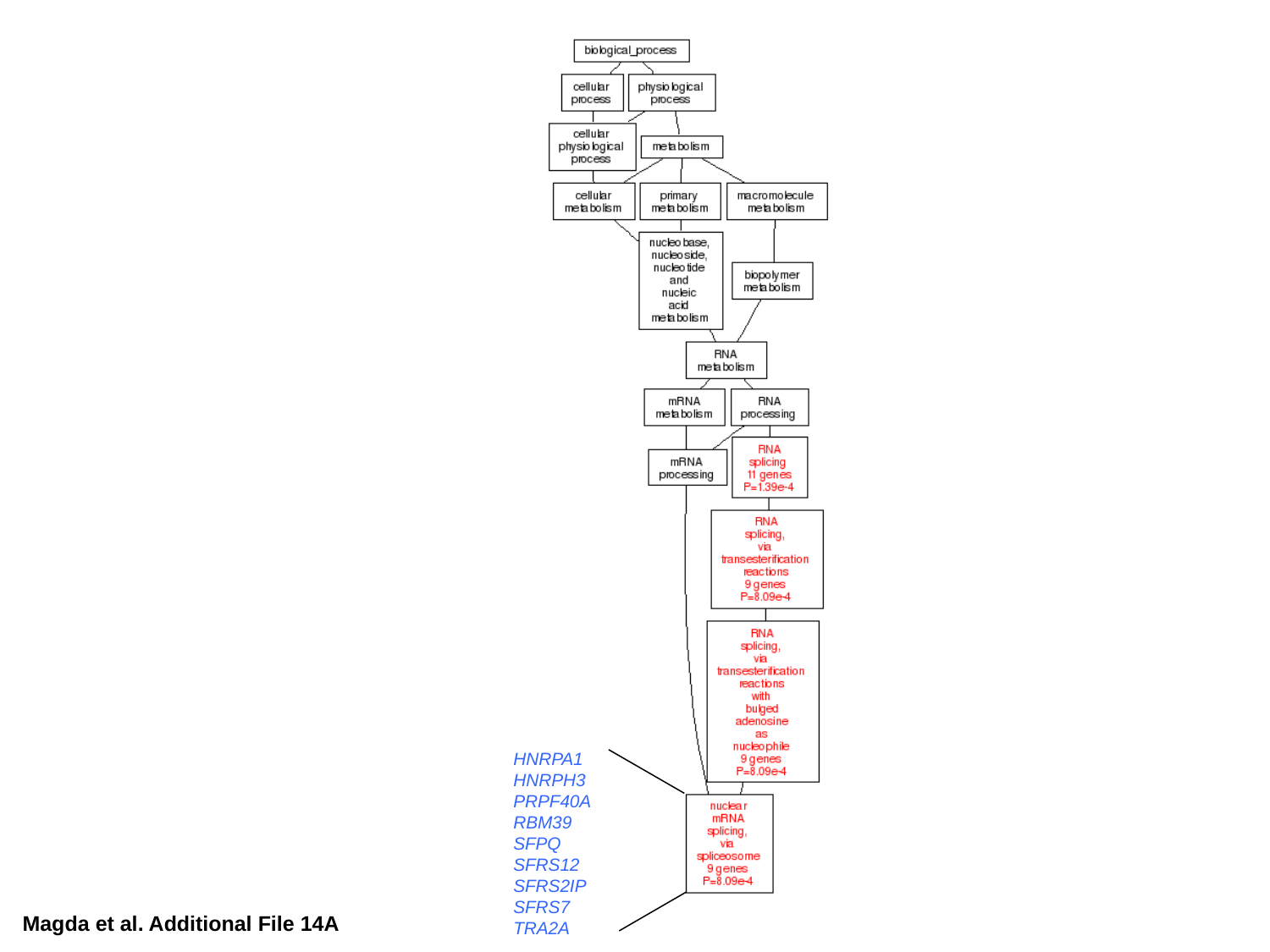

HNRPA1
HNRPH3
PRPF40A
RBM39
SFPQ
SFRS12
SFRS2IP
SFRS7
TRA2A
Magda et al. Additional File 14A

## Slide 3
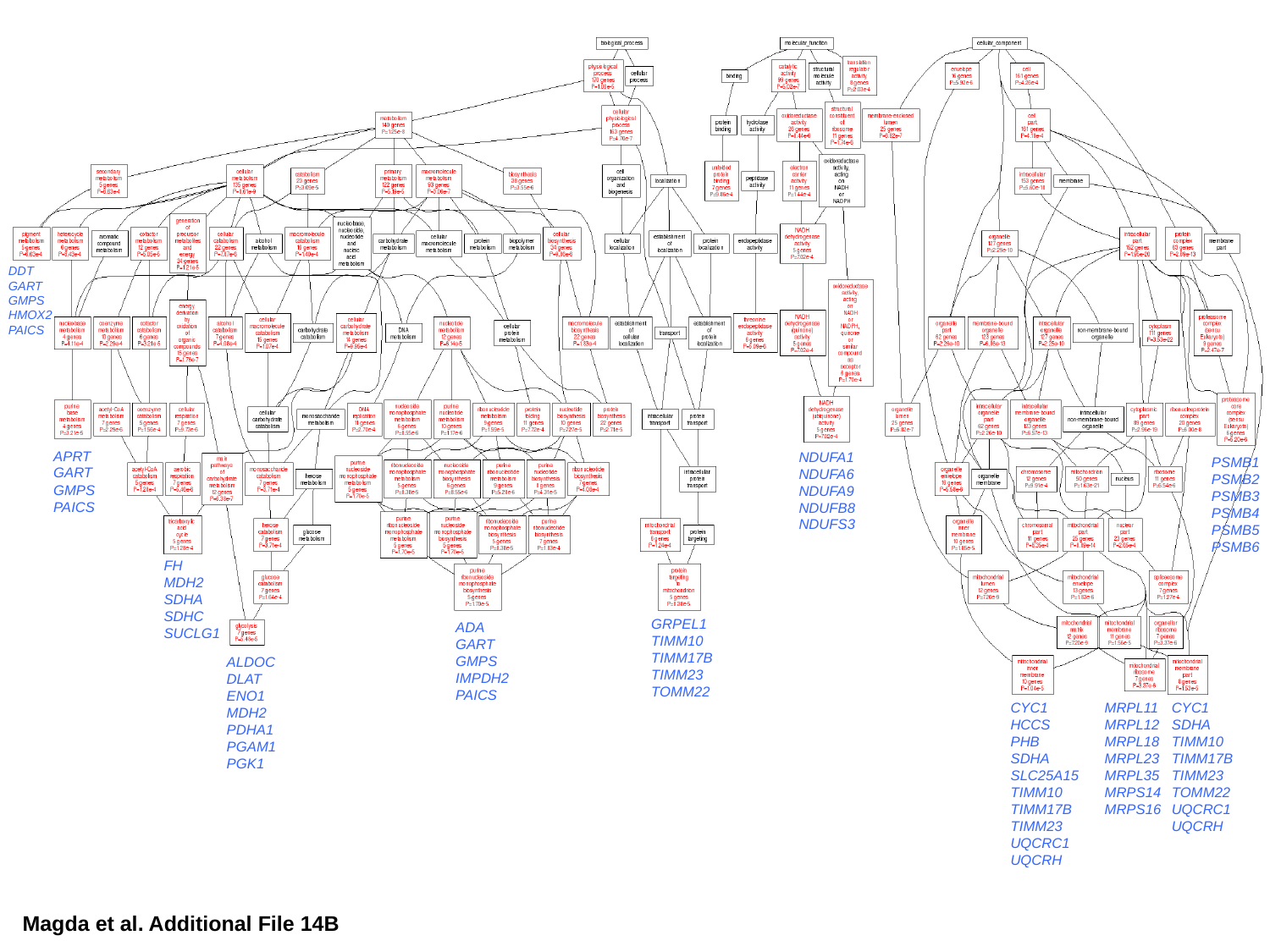

DDT
GART
GMPS
HMOX2
PAICS
APRT
GART
GMPS
PAICS
NDUFA1
NDUFA6
NDUFA9
NDUFB8
NDUFS3
PSMB1
PSMB2
PSMB3
PSMB4
PSMB5
PSMB6
FH
MDH2
SDHA
SDHC
SUCLG1
GRPEL1
TIMM10
TIMM17B
TIMM23
TOMM22
ADA
GART
GMPS
IMPDH2
PAICS
ALDOC
DLAT
ENO1
MDH2
PDHA1
PGAM1
PGK1
CYC1
HCCS
PHB
SDHA
SLC25A15
TIMM10
TIMM17B
TIMM23
UQCRC1
UQCRH
MRPL11
MRPL12
MRPL18
MRPL23
MRPL35
MRPS14
MRPS16
CYC1
SDHA
TIMM10
TIMM17B
TIMM23
TOMM22
UQCRC1
UQCRH
Magda et al. Additional File 14B
